# Cardiac regeneration and repair: the emerging mechanisms and therapeutic approaches

**DOI:** 10.1186/s43556-026-00504-6

**Published:** 2026-07-15

**Authors:** Yilong Li, Kexiao Zheng, Yinghuan Liu, Ran Li, Song Wang, Weicong Ye, Zilong Luo, Xiaohan Li, Zetong Tao, Jiahong Xia, Zifeng Zou, Yanglin Hao, Xi Zhang, Jie Wu

**Affiliations:** Department of Cardiovascular Surgery, Union Hospital, Tongji Medical College, Huazhong University of Science and Technology, Wuhan, 430022 China

**Keywords:** Heart regeneration, Cardiac repair, Immune microenvironment, Metabolic reprogramming, Signaling transduction, Clinical translation

## Abstract

Myocardial infarction, characterized by irreversible cardiomyocyte loss, progressive cardiac fibrosis and subsequent deteriorated heart function, constitute an intractable global health burden. Promoting endogenous cardiomyocyte proliferation to achieve functional cardiac regeneration represents an available foreground for combatting heart failure. Based on extensive preclinical evidence, researchers have characterized the cellular and molecular mechanisms governing cardiomyocyte proliferation and elucidated the modulatory influence of the metabolic factors and extracellular matrix. Insights from various studies have facilitated the development of regenerative approaches that reprogramming terminally differentiated cardiomyocytes toward a more metabolic plastic and fetal-like state, thereby enhancing the proliferative and reparative potential. Integration of metabolic and mechanical signals has emerged as a critical determinant of successful cardiomyocyte reactivation, providing a mechanistic rationale for combinatorial therapeutic strategies. Nevertheless, despite remarkable progression in mechanistic understanding and proof-of-concept studies, cardiac regeneration protocols advanced to robust clinical validation or pharmacal application have yet to be developed. This review provides a historical and biologically grounded synthesis of preclinical advances in cardiac repair, regeneration and cardiomyocyte reprogramming, with particular emphasis on translational feasibility and clinical applicability. Current strategies and preclinical trial outcomes are summarized to construct a readiness framework that maps the developmental maturity of each approach. Finally, the review highlights existing biological and technical barriers, as well as emerging opportunities poised to shape the future of cardiac regenerative medicine.

## Introduction

Cardiovascular disease remains the leading cause of human morbidity and mortality [1]. Among them, myocardial infarction (MI) continuously imposes a huge disease burden as it causes ventricular remodeling and further develop into heart failure [2]. Given the limited regenerative capacity of the adult human heart, MI results in a permanent deficit of functional cardiomyocytes [2–4]. Contemporary reperfusion strategies, guideline-directed medical therapy and device interventions mitigate myocardial remodeling and improve survival, but they do not restore cardiomyocyte loss [5–7]. Heart transplantation provides definitive replacement therapy for selected patients, yet its impact is constrained by donor scarcity and lifelong immunosuppression [8]. These realities sustain an emergency for regenerative myocardial repair [6].

Over the past decades, foundational work has revised long-standing assumptions about the regenerative ceiling of the adult mammalian heart [9]. Studies in highly regenerative species and neonatal mammals have revealed the inherent regenerative potential of cardiomyocytes, which is significantly suppressed in adulthood [10–12]. Similarly, cardiomyocytes in humans undergo lifelong renewal but decline steeply after infancy [9, 13]. Based on the above consensus, the core question in the field of cardiac regeneration was redefined as how to reactivate the dormant plasticity of cardiomyocytes in the mature and injured environments [13, 14]. Gradually, the focus of cardiac regeneration research has evolved from observational studies to the deeper investigation of underlying regeneration programs. Mechanistic dissection has further implicated multi-layered constraints that converge on cell-cycle control, including metabolic maturation, chromatin state, mechano-signaling and pro-regenerative niche. These insights have catalyzed an expanding therapeutic repertoire, spanning targeted modulation of metabolites, enzymes, transcriptional, epigenetic reprogramming, RNA and gene-based intervention to permissively orchestrate mechanics and inflammation [6]. Insights derived from these regenerative clues have significantly facilitated the process of translation of regeneration patterns from theory to clinical practice. However, clinical translation remains limited by fundamental hurdles such as delivery, specificity, durability, stability, and the challenge of scaling partial regeneration into functional recovery [15].

**Fig. 1:**
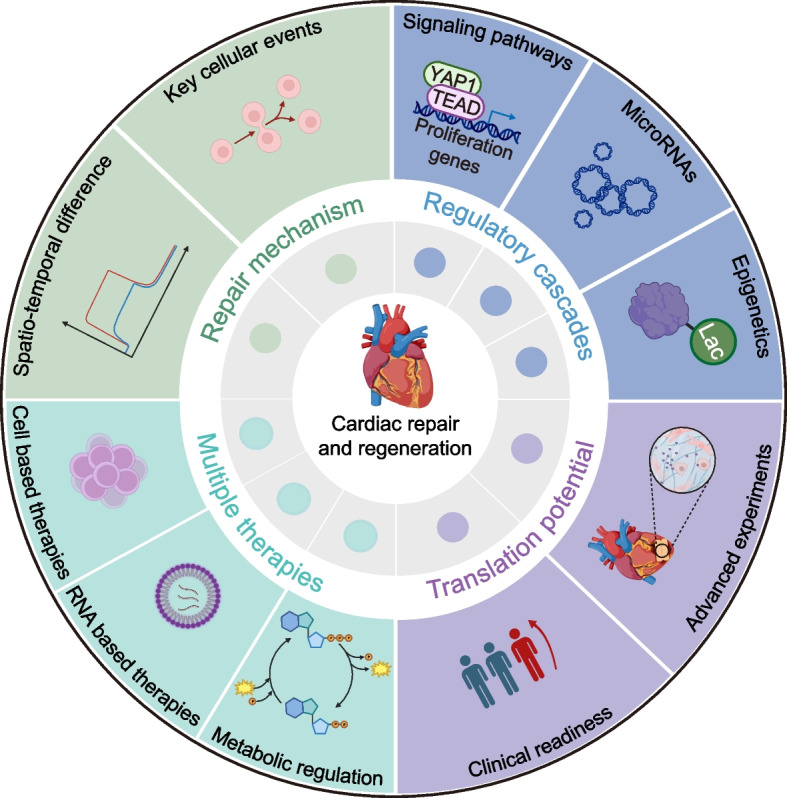
Integrated framework of cardiac repair and regeneration. The figure provides an integrated overview of the major themes discussed in this Review, linking endogenous repair mechanisms, regulatory cascades, multiple therapies and translation potential in cardiac repair and regeneration. (Figure was created with Biorender.com)

This review traces the historical development and major paradigm shifts in cardiac regeneration, synthesizes the core molecular pathways with their emerging therapeutic strategies, and assesses translational prospects from a pragmatic perspective. Finally, we proposed the priority directions for future research to bridge the gap between basic discoveries and clinically accessible cardiac regeneration therapies (Fig. 1).

## Endogenous mechanisms of cardiac regeneration and repair in progression

Research never stops at myocardial damage after repair as early as 1874, researchers began to study repair after injury to the frog’s heart. In 1937, pathologists discovered evidence of myocardial proliferation and hypertrophy from the myocardial sections of human children [16]. In 1968, Soviet scientists discovered evidence of increased mitosis of cardiac muscle cells after myocardial infarction in a mouse model [17]. However, these morphological observational studies do not address true regeneration evidence. The history of cardiac regeneration research has undergone a fundamental reassessment from an inherent concept of "terminal differentiation" to the recognition that cardiomyocytes have a limited but mechanistically exploitable capacity for regeneration. Early foundational evidence came from classic experiments in lower vertebrates. Studies of urodele amphibians in 1974 revealed that adult newt ventricles can regenerate substantial portions of myocardium after apical resection, restoring muscle tissue rather than forming a permanent scar [18]. Subsequent work in newt myocardial explants demonstrated that mature cardiomyocytes can re-enter the cell cycle and undergo extensive structural remodeling in response to regenerative cues, reinforcing the concept of life-long regenerative competence in certain vertebrates [19].

A major conceptual turning point came with the establishment of a standardized zebrafish apical-resection model in 2002. Poss et al. Reported that the removal of ~20% of the ventricle is followed by near-complete structural and functional restoration within weeks [20]. Genetic lineage-tracing studies revealed that regenerated myocardium originates from pre-existing cardiomyocytes rather than from a distinct stem-cell pool [21, 22]. Comparative work across teleost and amphibian species subsequently delineated a continuum of regenerative phenotypes and highlighted coordinated changes in the state of cardiomyocytes, vascular remodeling, immune-cell dynamics and extracellular-matrix organization as core determinants of regenerative outcomes [23].

These comparative insights motivated the search for analogous regenerative processes in mammals. The demonstration that neonatal mice (P1) regenerate myocardium after apical resection, whereas P7 hearts heal by fibrosis, identified a brief postnatal regenerative window in which injury elicits cardiac regeneration rather than scar formation [9]. Lineage-tracing and molecular studies confirmed that newly formed myocardium in P1 mice arises predominantly from pre-existing cardiomyocytes, mirroring the regenerative mechanism observed in zebrafish and other lower vertebrates [9]. Work in large animals and human infants supported the broader principle that mammalian cardiac regenerative capacity is developmentally restricted: Mollova et al. quantified robust cardiomyocyte proliferation in neonatal human hearts with a rapid decline after infancy, indicating that the number of cardiomyocytes is largely set early in life [24].

The question of whether adult humans retain a measurable degree of cardiomyocyte regeneration was subsequently addressed through C14 birth-dating of purified cardiomyocyte nuclei. This approach showed that human cardiomyocytes are indeed replaced throughout life, albeit at low annual rates that decline with age [25], and that non-myocyte lineages, particularly endothelial and mesenchymal cells exhibit substantially greater turnover than cardiomyocytes do [26]. Recent work in patients with end-stage heart failure and left-ventricular assist device (LVAD) support has further refined this picture. Derks et al. demonstrated that cardiomyocyte generation is profoundly suppressed in failing hearts but can increase several-fold during mechanical unloading and reverse remodeling, revealing a latent cardiomyocyte regeneration potential that remains exploitable in human heart disease [27].

A parallel line of research in the late 1990 s and early 2000 s focused on the concept of adult cardiac stem or progenitor cells, following reports that c-kit⁺ cells or bone-marrow-derived cells might generate new myocardium [28, 29]. This paradigm briefly shaped the field and motivated numerous clinical trials [30]. However, subsequent genetic lineage-tracing and rigorous fate-mapping studies demonstrated that these populations make negligible contributions to new cardiomyocytes and that the clinical benefits of cell therapy are modest and largely paracrine [31–33]. As evidence accumulated, the cardiac stem cell has model lost prominence, and regeneration research has shifted back toward cardiomyocyte-centered and microenvironment-based mechanisms.

Taken together, these experimental advances support a working model in which cardiac repair capacity reflects a conserved but progressively developed regenerative program shaped by species identity, developmental stage cardiac microenvironment and disease state, providing the conceptual foundation for the fate-mapping, cardiomyocyte plasticity and extracellular-matrix mechanisms examined in the following sections.

**Fig. 2:**
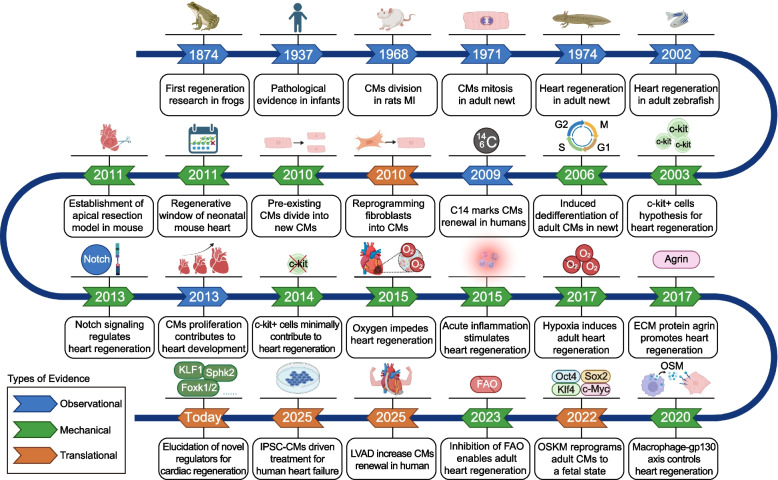
Conceptual advances in cardiac regeneration research. The timeline summarizes landmark discoveries that shaped the field of cardiac regeneration, from early observational evidence across species to mechanistic insights into cardiomyocyte renewal, hypoxia, inflammation, extracellular matrix regulation, metabolic reprogramming and cell-state plasticity. The color-coded labels distinguish observational, mechanistic and translational evidence, highlighting the progressive transition from biological discovery toward therapeutic exploration. (Figure was created with Biorender.com)

Recent decades have seen remarkable theoretical advances in cardiac regeneration research (Fig. 2). Early controversies about the existence, origin, and extent of cardiomyocyte regeneration have gradually given way to a more coherent framework supported by evidence from lineage tracing, genetic fate mapping, and comparative regeneration models. The synthesis of these studies has resulted in some widely accepted principles that form the novel basis of this field. Among them, two concepts stand out as central and functionally inseparable elements: the cellular origin of regenerating cardiomyocytes and the necessity for cardiomyocytes to regain a plastic state in to regenerate. These consensus views not only resolve long-standing debates but also suggest that cardiac regeneration is a problem of cell state control within the injured heart, thus providing a unified framework for subsequent mechanistic and therapeutic studies.

### Cardiomyocyte plasticity and renewal following injury

**Fig. 3:**
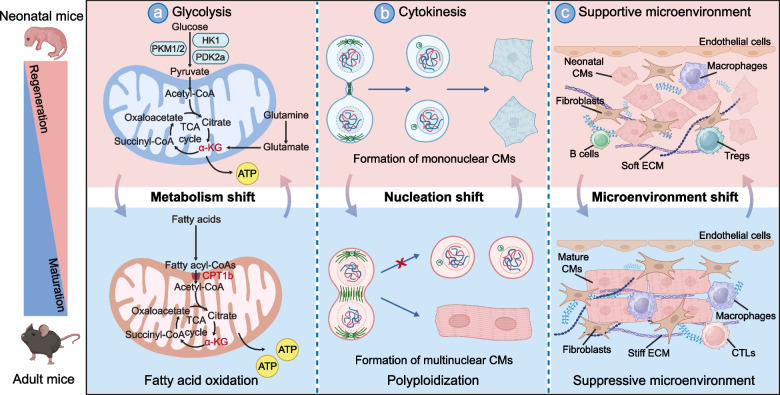
The metabolic and molecular basis of cardiac regeneration. Cardiomyocyte proliferation declines postnatally, coinciding with the rapid progression of cardiomyocyte maturation. Mechanistically, three significant molecular events can be identified for this progress. **a** Metabolism shift. Mature cardiomyocytes undergo a transition in energy supply pattern from glucose oxidation towards fatty acid oxidation metabolism and oxidative phosphorylation. **b** Nucleation shift. During postnatal maturation, mammalian cardiomyocytes undergo genomic duplication to form polyploid cells, at the cost of impaired cytokinesis. In contrast, cardiomyocytes capable of cytokinesis tend to divide and maintain proliferation capacity. **c** Microenvironment shift. Postnatal cardiac development is characterized by a significant increase of non-cardiomyocyte cells such as macrophages and T cells. This change is paralleled by a shift of inflammation response within cardiac microenvironment, which imped cardiac regeneration. (Figure was created with Biorender.com)

Across species and developmental stages, cardiac regenerative capacity is largely determined by whether mature cardiomyocytes can re-enter the cell cycle and complete productive cytokinesis after injury. This property is not dictated by cardiomyocytes in isolation but emerges from a tightly coupled metabolic and microenvironmental network that includes cardiomyocytes, fibroblasts, endothelial cells and immune cells [34–40]. Within this network, distinct metabolic activities shape the proliferative potential of cardiomyocyte. For example, sphingolipid metabolism influences cardiomyocyte maturation and polyploidization while simultaneously modulating fibroblast activation and fibrogenesis within the myocardial niche [41]. In neonatal mouse hearts, macrophages undergoing efferocytosis encode arachidonic acid metabolism and, in so doing, activate pro-regenerative signaling pathways in neighboring cardiomyocytes [42]. At the level of the cardiomyocyte itself, metabolic enzymes directly interact with the cell-cycle machinery: PKM2, a central regulator of glycolytic flux, promotes G1–S progression by controlling cyclin D expression through the WNT/β-catenin pathway [43]. Taken together, these observations indicate that metabolic state is not a passive readout of cellular activity, but rather an active determinant of cardiomyocyte cell-cycle competence (Fig. 3).

During mammalian development, the rise and subsequent loss of regenerative capacity closely track a transition from a fetal, glycolytic program to a postnatal, fatty-acid-oxidation (FAO)–dominated oxidative program [44, 45]. Fetal and early neonatal cardiomyocytes function in a relatively hypoxic environment and rely predominantly on glycolysis for ATP production [46]. The proliferation permissive state is characterized by immature mitochondria and low oxidative stress. In mice, such a metabolic configuration supports robust regeneration after apex resection, cryoinjury or myocardial infarction during the first postnatal week. Beyond postnatal day 7, regenerative capacity declines sharply, as a rapid increase in FAO, oxidative phosphorylation and mitochondrial biogenesis occur [9, 45]. Although this metabolic maturation is essential for meeting the contractile demands of the adult heart, it is tightly linked to stable cell-cycle exit. Glycolysis supports the biosynthetic flux required for growth and division, whereas FAO provides ATP efficiently but poorly supports anabolic expansion. The postnatal metabolic “switching” is therefore widely regarded as a central mechanism underlying the closure of the regenerative window.

Additional postnatal events further narrow the pool of cardiomyocytes capable of proliferation. Polyploidization and multinucleation, primarily stemming from cytokinesis failure, are hallmarks of postnatal mammalian cardiomyocytes [47]. These processes are postulated to sustain contractile function in response to increasing hemodynamic loads [48]. However, these processes inevitably reduce the fraction of mononuclear cardiomyocytes that retain the structural capacity for true cell division. Clinical observations add another layer to this picture: mechanical unloading with a LVAD can reduce cardiomyocyte DNA content and increase indices of cell-cycle activity, suggesting that mechanical stress and metabolic configuration act together to drive a developmental trajectory from a proliferative, plastic phenotype to a terminally differentiated, contractile one [49].

In contrast, in highly regenerative species such as adult zebrafish, injury elicits fundamentally different responses. Following ventricular resection, zebrafish regenerate lost myocardium almost completely through dedifferentiation, proliferation, migration and redifferentiation of pre-existing cardiomyocytes [22]. This cellular sequence is accompanied by characteristic metabolic reprogramming, such as reduced expression of mitochondrial genes and succinate dehydrogenase activity, upregulation of glycolytic enzymes (including HK1, PKM1/2 and PDK2a), and increased glucose uptake and accumulation of metabolites such as α-ketoglutarate, which serve as cofactors for epigenetic enzymes [50, 51]. Pharmacological inhibition of glycolysis with 2-deoxyglucose suppresses proliferation-associated transcriptional programs and impairs regeneration. These findings point to a conserved metabolic reprogramming–epigenetic–transcriptional axis that links metabolic flexibility to cardiomyocyte regenerative competence [50].

Integrating developmental and cross-species data, an increasingly influential concept is that the adult mammalian heart harbors a small population of “proliferation-competent” cardiomyocytes. These cells are structurally mature but retain elements of fetal-like metabolic, transcriptional and cell-cycle plasticity, rendering them more responsive to injury-induced dedifferentiation and cell-cycle re-entry. Using a hypoxia fate-mapping strategy, Kimura et al. identified a rare population of hypoxia-traced cardiomyocytes in the adult mouse heart that displayed neonatal-like characteristics, such as smaller cell size and mononucleation, together with signs of cell-cycle activity, supporting the possibility that proliferation-competent cardiomyocytes are enriched in hypoxic myocardial niches [52]. Thus, defining the origin, frequency and behavior of this subset is necessary and continues to require precise genetic fate-mapping approaches, which have substantially reshaped long-standing views on the cellular source of new cardiomyocytes.

### The role of Cardiac Progenitor Cells (CPCs) in cardiac repair

The notion of cardiac progenitor cells (CPCs) is firmly rooted in embryonic heart development. During cardiogenesis, progenitor populations that marked by Isl1, Nkx2.5 or KDR give rise to cardiomyocytes, endothelial cells and smooth muscle cells across the primary and secondary heart fields, placing CPCs at the center of early heart formation [53–55]. Single-cell and clonal analyses combined with genetic lineage tracing demonstrate that these embryonic progenitors possess robust multilineage potential. Whether comparable progenitors in the adult heart act as meaningful sources of new cardiomyocytes has been fundamentally reexamined [31].

Multiple CPC-like populations including c-kit⁺ cells, Sca-1⁺ cells and cardiosphere-derived cells has been isolated from the adult myocardium, [33, 56]. Early studies interpreted these populations as putative “cardiac stem cells” with the capacity to regenerate cardiomyocytes after injury. However, cell-specific fate mapping experiments have consistently challenged this view. In vivo, c-kit⁺ and other CPC-like lineages contribute minimally to new cardiomyocytes and instead predominantly give rise to endothelial and interstitial cells [26, 31]. These convergent data support a paradigm in which adult CPC-like cells are more accurately regarded as modulators of paracrine signaling or matrix remodeling in the cardiac microenvironment than as a primary endogenous source of new cardiomyocytes [33, 57]. In parallel, lineage-tracing studies focused on mature cardiomyocytes have revealed a different picture of regeneration. In zebrafish, neonatal mammals and selected adult rodent models, the overwhelming majority of newly formed cardiomyocytes can be traced back to pre-existing cardiomyocytes that undergo injury-induced dedifferentiation, cell-cycle re-entry and redifferentiation [9, 22, 58]. In neonatal mice, this mechanism underlies the transient regenerative window after birth; in adult zebrafish, it supports near-complete structural and functional recovery after substantial ventricular injury [9, 22]. These observations indicate that mature cardiomyocytes rather than CPCs constitute the primary endogenous source of new myocardium in the adult mammalian heart.

The recognition that endogenous CPC-like cells have limited myogenic output has shifted attention toward the use of pluripotent stem cell (PSC)–derived cardiovascular progenitors (PSC-CPCs) as exogenous platforms for structural repair. Human ESCs or iPSC-derived cardiac and cardiovascular progenitors can be produced at scale, maintained at a defined cardiogenic mesoderm stage and subsequently directed towards cardiomyocyte, endothelial and smooth muscle fates under controlled differentiation conditions [59–61]. In a landmark porcine myocardial infarction model, transplantation of PSC-derived committed cardiac progenitors resulted in stable engraftment, differentiation into cardiac and vascular lineages, reduced scar burden and improved ventricular function [62, 63]. Related work using porcine CPCs engineered to overexpress IGF-1 and HGF in sub-acute infarct models further supports the idea that progenitor populations can beneficially modulate post-infarction remodeling and function, although in these settings the dominant effects are probably paracrine rather than directly remuscularization [62, 63].

These preclinical advances have begun to inform early-phase clinical experience. In the ESCORT program, Menasché et al. generated clinical-grade hESC-derived cardiovascular progenitors, embedded them in a fibrin scaffold and applied the resulting epicardial patch to patients with advanced ischemic heart failure undergoing surgical revascularization [64, 65]. This first-in-human case report and subsequent phase I trial demonstrated technical feasibility and an acceptable short- to mid-term safety profile, with signs of stabilization or modest improvement in left ventricular function in selected patients, although these studies were not designed to assess efficacy [64, 65].

Taken together, the available data support a nuanced view: adult CPC-like cells primarily act as regulators of the reparative microenvironment, mature cardiomyocytes provide the main endogenous source of new muscle, and PSC-derived CPCs are emerging as a central component of exogenous strategies that aim to rebuild the myocardium and vasculature. Future regenerative protocols are likely to deploy PSC-CPCs in combination with optimized delivery routes, bioengineered matrices, and immune-modulatory or pro-proliferative cues, with the dual goals of maximizing engraftment and translating structural benefits into durable clinical improvement.

### The Extracellular Matrix (ECM) and the dynamic scar

The extracellular matrix (ECM) is increasingly recognized as a dynamic mechanic system that controls the proliferative competence of cardiomyocyte during development and after injury [66–68]. The mechanical landscape of the cardiac microenvironment undergoes dynamic remodeling during development and pathology [69, 70]. Specifically, cardiac ECM phenotype progressively increases with developmental maturation, whereas pathological insults such as MI induce significant matrix stiffening in the injury zone [70]. Notably, this temporal alteration in mechanical rigidity inversely correlates with the loss of cardiomyocyte regenerative capacity. Notari et al. pinpointed this critical window in a neonatal mouse model: while 1-day-old mice mount a robust regenerative response following apical resection, this capacity decreases precipitously within 48 h, shifting the repair mechanism in 2-day old mice toward fibrotic scarring [69]. Transcriptomic comparison of whole hearts from P1 and P2 mice revealed that the majority of differentially expressed transcripts encode components of the ECM and cytoskeleton. These findings suggest that local microenvironmental stretch acts as a determinant of cardiac regenerative potential. Indeed, experimentally softening the ECM in P3 mice successfully reactivated the regenerative program following apical resection [69]. Although the extent of regenerative benefit varies across different experimental models, converging evidence from mammalian studies confirms the consensus that the mechanical properties of the ECM are fundamental regulators controlling cardiac regeneration.

During the neonatal period, the myocardium is embedded in ECM enriched with regenerative cues such as Agrin, a heparan sulfate proteoglycan that activates ERK and liberates YAP from the dystrophin–glycoprotein complex (DGC) to promote cardiomyocyte cell cycle re-entry [71, 72]. As the heart matures, progressive collagen deposition [69], enhanced lysyl oxidase-dependent cross-linking [73], and consolidation of membrane adhesion complexes collectively restrain YAP nuclear activity and close the regenerative window [74–76]. These developmental transitions position the ECM as a central determinant of cardiomyocyte competency, converting a permissive neonatal niche into an increasingly rigid, YAP-repressive adult microenvironment. Following myocardial injury, scar formation represents not a static endpoint but a staged and active remodeling process that re-wires ECM–cytoskeletal–YAP signaling [77]. Early provisional matrix and inflammatory ECM transiently support cytoskeletal plasticity, but as collagen I/III accumulates and cross-links, the maturing scar imposes sustained mechanical tension that promotes YAP activation in fibroblasts while simultaneously suppressing YAP-dependent cell-cycle activity in cardiomyocytes [72].

Recent work has revealed the cytoskeletal mechanisms that mediate this divergence: after infarction, microtubule expansion and sirtuin downregulation drive YAP acetylation and sequestration on TUBA4A polymers, preventing its nuclear translocation and blocking regeneration [77]. Together with evidence that actomyosin tension modulates YAP nuclear access, these findings establish the cytoskeleton as the critical conduit through which a dynamic scar converts ECM remodeling into durable repression of cardiomyocyte proliferation [77]. Emerging evidence from multiomics and functional studies underscores the critical role of a plastic, juvenile ECM in defining the neonatal regenerative window. Through proteomic profiling, Versican was identified as a key driver of this process. Notably, Versican promotes regeneration by specifically stimulating cardiomyocyte proliferation and has been shown to restore function in adult mouse models of permanent occlusion and I/R injury [78]. With respect to the enzymatic regulation of matrix remodeling, Mmp14b and its enhancer (Mmp14b-*enh1*) has been identified as upstream mechanisms that maintains the bioavailability of Agrin to ensure the plasticity of the neonatal ECM [79]. Additionally, cross-talk between cell types plays a vital role, as evidenced by findings that endocardial secretion of Lyz2 remodels the local ECM to modulate regenerative outcomes [80]. Together, these insights suggest that recapitulating an embryonic-like ECM niche via targeted intervention represents a viable strategy for adult heart repair.

**Fig. 4:**
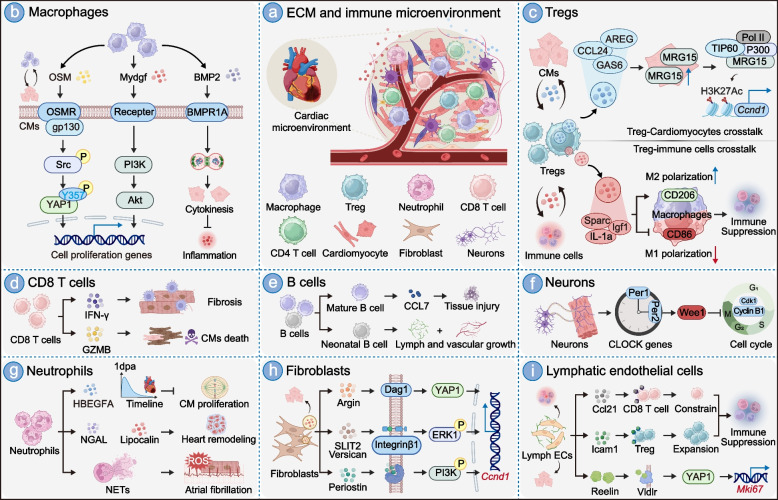
The role of cardiac microenvironment in heart repair and regeneration. **a** Myocardial infarction induces a dynamic and plastic cardiac microenvironment that coordinates the reparative response and shapes regenerative potential. **b** Macrophages promote cardiomyocyte proliferation through paracrine pathways, including OSM–gp130, MYDGF–PI3K and BMP2–BMPR1A signaling. **c** Regulatory T cells modulate cardiac repair through direct Treg–cardiomyocyte crosstalk and indirect Treg–immune cell interactions, thereby promoting an immunosuppressive niche that favors tissue repair. **d** CD8 + T cells reshape the cardiac microenvironment by secreting IFN-γ and GZMB, thereby accelerating fibrosis and clinical death risk. **e** B cells at distinct developmental stages exert coordinated and context-dependent effects on the repair response after myocardial infarction. **f** Neuron-Cardiomyocyte crosstalk plays a vital role in cardiac repair by reactivating CLOCK genes. **g** Neutrophils participate in the early repair response through a transient post-infarction peak that may support cardiomyocyte proliferation; however, excessive or persistent neutrophil activity, including secretion of NGAL/lipocalin-2 and formation of neutrophil extracellular traps (NETs) aggravate cardiac injury. **h** Fibroblasts regulate cardiomyocyte growth and regenerative responses through extracellular matrix and paracrine factors, including Agrin, Versican, SLIT2 and Periostin. **i** Lymphatic endothelial cells support cardiac regeneration through paracrine mediators such as Ccl21, Icam1 and Reelin. (Figure was created with Biorender.com)

These insights have led to the repositioning of the ECM from a passive bystander to a tractable therapeutic target capable of re-opening latent regenerative programs in the adult heart. Neonatal-like ECM cues, particularly Agrin, can attenuate the inhibitory adult scar environment and re-induce cardiomyocyte cycling in multiple models of injury. Mechanical reprogramming strategies, including inhibition of collagen cross-linking, softening of the infarct matrix, and deployment of decellularized or biomimetic matrices, have been shown to increase YAP nuclear activity and limit pathological fibrosis. Moreover, targeting cytoskeletal checkpoints such as YAP acetylation or microtubule-mediated sequestration offers a mechanistically precise means to counteract scar-imposed proliferative arrest. Together, these approaches support a unifying model in which modulating the ECM and its downstream cytoskeletal signaling can shift the balance from irreversible scarring toward partial restoration of regenerative capacity (Fig. 4).

### The immune microenvironment following injury

The reparative potential of the injured heart extends beyond the intrinsic proliferative capacity of cardiomyocytes and is critically governed by the local immune microenvironment. Rather than a mere secondary response to necrosis, immune activation acts as a primary determinant, directing the injured tissue toward either functional regeneration or adverse remodeling. In regenerative models, the injury-induced inflammation is spatiotemporally restricted and rapidly undergoes resolution. This transient inflammatory phase facilitates the efficient clearance of necrotic debris, promotes angiogenesis, and preserves ECM plasticity, ultimately establishing a permissive niche for cardiomyocyte cell-cycle re-entry. Conversely, the adult mammalian heart exhibits a prolonged, non-resolving inflammatory response. Persistent pro-inflammatory leukocyte infiltration and dysregulated ECM turnover actively impede functional restoration, driving the myocardium toward progressive adverse remodeling and permanent fibrosis (Fig. 4).

Neonatal cardiac regeneration models directly validate this microenvironmental dependency. In murine models, macrophage depletion following myocardial infarction markedly inhibit regeneration and impairs neovascularization, establishing innate immune cells as integral components of the regenerative program rather than passive scavengers [37]. Subsequent lineage-tracing studies established cellular origin as a fundamental determinant of these distinct reparative outcomes [35]. Embryonic-derived resident cardiac macrophages expand to coordinate the neonatal regenerative response, whereas monocyte-derived inflammatory macrophages rapidly infiltrate and overwhelm the injured adult heart. Consistent with these distinct lineage roles, blocking monocyte influx in adult models preserves the resident macrophage pool, attenuates inflammation, and improves tissue repair, establishing macrophage origin as a critical determinant of functional recovery.

Beyond cellular origin, the temporal dynamics of the immune response critically determine whether the injured heart undergoes regeneration or fibrotic scarring. Comparative studies in zebrafish and medaka revealed that the limited regenerative capacity of the medaka heart stems from delayed macrophage recruitment and impaired neutrophil clearance, which collectively perpetuate a profibrotic milieu [81]. In zebrafish, however, macrophages infiltrate the injury site at the precise temporal juncture required to coordinate debris clearance and scar regression. This principle translates to adult mammalian models through the biphasic monocyte response following myocardial infarction. The sequential transition from early Ly6C^high^ proinflammatory monocytes to late Ly6C^low^ reparative monocytes serves as a critical determinant of healing quality [82]. Ultimately, regenerative failure is driven less by the initial intensity of inflammation than by a prolonged proinflammatory state, impaired lineage transitions, and failing resolution mechanisms.

As central orchestrators of this reparative network, macrophages cannot be strictly defined as either beneficial or detrimental; instead, their functional polarization is continuously shaped by the local microenvironment. Single-cell transcriptomic profiling in zebrafish has resolved distinct, regeneration-associated resident macrophage subpopulations required for coordinating neovascularization, cardiomyocyte survival, and ECM turnover [83]. Conversely, in adult non-regenerative repair, the macrophage pool shifts heavily toward monocyte-derived, CCR2 + inflammatory subsets. In pressure overload models, the infiltration of these CCR2 + macrophages actively drive pathological ventricular remodeling and dysfunction. Furthermore, distinct CCR2 − and CCR2 + resident subsets differentially orchestrate subsequent monocyte recruitment and fate specification, illustrating how the baseline architecture of the resident niche dictates the ensuing inflammatory cascade [84]. Recent research has demonstrated that following cardiac injury, macrophages utilize Toll-like receptor 4 (TLR4) to engage CLU, the protein secreted by border-zone specific Clu + cardiomyocytes. This ligand-receptor interaction triggers Cpt1a-dependent FAO within the macrophages, effectively attenuating inflammation and driving their polarization toward a reparative phenotype. Subsequently, these polarized macrophages secrete bone morphogenetic protein 2 (BMP2) to reciprocally activate bone morphogenetic protein receptor type 1 A (BMPR1A) signaling in CMs, ultimately driving CM proliferation and tissue regeneration.

Unlike the lineage dependency of macrophages, the contribution of neutrophils is strictly governed by temporal windows. During the acute phase of injury, rapid neutrophil influx is necessary to clear necrotic debris and prime the subsequent repair cascade. However, when neutrophils exhibit prolonged retention or delayed clearance, their function transitions from initiating repair to sustaining damage. Persistent neutrophils exacerbate cardiomyocyte death and anchor a proinflammatory macrophage stage via the continuous release of proteases, reactive oxygen species (ROS), and neutrophil extracellular traps (NETs) [85]. Mechanistically, overexuberant neutrophil signaling directly amplifies post-infarction apoptosis, whereas promoting timely neutrophil apoptosis and subsequent efferocytosis facilitates pro-resolving macrophage polarization. Recent studies have found that neutrophils can promote epicardial regeneration response by releasing signals such as heparin-binding EGF-like growth factor a (hbegfa) during zebrafish cardiac regeneration, indicating that neutrophils may even be one of the necessary initiators at the early stage of regeneration [86]. Functional regeneration, therefore, demands a strictly self-limiting neutrophilic response rather than sustained cytotoxic infiltration.

Beyond innate immune kinetics, the overarching immunological polarization profoundly influences the reparative outcome. Neonatal immunity exhibits a developmental skew toward type 2 responses, establishing an immunological niche critical for regeneration [87]. The loss of IL-4 and IL-13 in neonatal mice shifts the local microenvironment toward an adult-like, proinflammatory state, thereby impairing functional recovery after ischemic injury. Conversely, exogenous administration of these cytokines rescues both the immune profile and cardiac function, while myeloid-specific disruption of IL-4Rα signaling directly compromises regeneration. These findings demonstrate that type 2 cytokine programs actively sustain a macrophage polarization state conducive to a regenerative niche, whereas injured adult hearts default to a type 1 dominant network that actively impedes tissue integration.

Within the adaptive immune compartment, Tregs are classically defined as a reparative population; however, this protective identity is highly susceptible to environmental reprogramming. In neonatal models, Tregs directly promote cardiomyocyte proliferation via paracrine signaling and mitigated fibrosis, confirming that their regenerative capacity extends beyond canonical immunosuppression [88]. Conversely, prolonged pathological stress in ischemic cardiomyopathy and heart failure induces maladaptive Treg reprogramming, driving a phenotypic transition toward proinflammatory and anti-angiogenic states that promote adverse ventricular remodeling [89]. Consequently, the loss of immune protection in adult disease stems primarily from the maladaptive reprogramming of these subsets rather than their absolute numerical decline.

Distinct from the plasticity of Tregs, CD8 + T cells and specific effector subsets serve as intrinsically pathogenic mediators of adult fibrotic remodeling. Cross-presenting dendritic cells (DCs) exacerbate post-ischemic damage by capturing necrotic myocardial antigens and activating cytotoxic CD8 + T cells, sustaining immune-mediated tissue injury [90]. Concurrently, ischemia-recruited CD8 + T cells release granzyme B to directly induce cardiomyocyte apoptosis and drive adverse remodeling [91]. Targeted depletion of these cells robustly attenuates tissue damage, limits fibrotic transcription, and improves cardiac function. Collectively, these mechanisms delineate an adaptive immune-mediated axis of antigen presentation and cytotoxic injury that actively exacerbates adverse ventricular remodeling in the adult heart.

The role of B lymphocytes is similarly dictated by developmental stage, undergoing a stark reversal from pro-regenerative to anti-reparative in adulthood. Neonatal cardiac-resident B cells exhibit a distinct pro-angiogenic transcriptional profile and are required for cardiomyocyte proliferation [92]. In adult myocardial infarction, however, mature B cells actively drive the mobilization of Ly6C^high^ monocytes from the bone marrow via CCL7 secretion, severely aggravating local inflammation and functional deterioration [82]. Consequently, genetic or antibody-mediated depletion of mature B cells effectively disrupts this axis and improves structural outcomes. These opposing functions emphasize that adult non-regenerative repair is not characterized by a simple absence of regenerative effectors, but by the pathological rewiring of immune networks that actively enforce scar stabilization.

In conclusion, the balance between cardiac regeneration and fibrotic repair is critically governed by the spatiotemporal dynamics and lineage specificity of the local immune response. A regenerative microenvironment necessitates the rapid resolution of inflammation, a process coordinated by tissue-resident macrophages and reparative type 2/Treg-mediated adaptive immunity. Conversely, the adult mammalian myocardium is predisposed to maladaptive remodeling, propagated by unresolved monocyte infiltration, impaired efferocytosis, and the deleterious crosstalk between mature T and B lymphocytes. Ultimately, the structural and functional outcome following injury depends not merely on the magnitude of immune activation, but rather on the precise temporal orchestration of these immune networks and their capacity to transition the myocardial niche from a pro-fibrotic state toward functional restoration.

## Reactivation of the cardiac intrinsic repair programs

### Metabolic patterns and reprogramming in the injured heart

Metabolic regulation of cardiac regeneration has been validated across multiple species and injury models. These studies demonstrate that metabolism plays a conserved and causal role in determining regenerative competence.

In adult zebrafish subjected to cryoinjury, border-zone cardiomyocytes undergo a shift toward glycolysis. Pharmacological inhibition of glycolysis with 2-deoxyglucose markedly reduces cardiomyocyte proliferation and impairs regeneration [51, 93]. These findings indicate that enhanced glucose metabolism is required for effective myocardial regeneration. Similar principles apply in mammals that in neonatal and adult mouse hearts, modulation of glucose utilization influences cardiomyocyte cell-cycle activity. Regulation of mitochondrial pyruvate entry through nodes such as GLUT1 and the PDK4–PDH axis alters post-injury repair outcomes [94, 95]. In adult mouse ischemia–reperfusion models, direct inhibition of fatty acid oxidation promotes regeneration. Suppression of CPT1b-dependent pathways increases α-ketoglutarate availability. This enhances KDM5-mediated epigenetic remodeling. As a result, cardiomyocyte proliferation increases and structural and functional recovery improve [50]. Evidence is also emerging from large-animal systems. In porcine myocardial infarction models, transient enhancement of PKM2 expression has been achieved using modified mRNA delivery. This intervention improves ventricular remodeling and cardiac function. It is accompanied by increased regenerative molecular and cellular signatures [96]. Studies based on myocardial specimens from patients with congenital heart disease, together with investigations in iPSC-CMs indicate that human cardiac regeneration and regeneration-associated plasticity are accompanied by a defined metabolic shift [45, 97].

Taken together, these cross-species and cross-model findings support a unifying concept. Metabolic state is not merely a correlate of regeneration. It is an upstream and experimentally manipulable determinant of cardiomyocyte plasticity and regenerative capacity (Table 1).

**Table 1:** Cross-species evidence for the metabolic regulation of cardiac regeneration

Species	Animal model	Metabolic targets	Treatments	Readouts	Evidence type	TRL (1–9)	Reference
Zebrafish	Ventricular resection/cryoinjury	Glucose metabolism (glycolysis)	PDK3 overexpression (genetic or metabolic interventions)	Border-zone CM proliferation and cell cycle programs	Genetic manipulation; pharmacologic evidence	3	[51]
Mouse (Neonatal)	Cryoinjury (αMHC-hGLUT1 transgenic mice)	Glucose metabolism (glycolysis)	CM specific GLUT1 overexpression	Reduced fibrosis; improved structural/functional repair; benefit abrogated by inhibiting nucleotide synthesis	Genetic manipulation; pharmacologic evidence	4	[94]
	Neonatal/adult injury comparison	Lipid signaling	Immune–lipid metabolic pathway rewiring	Promotes neonatal CM glycolysis and cell-cycle programs	Cross-age comparison validation	4	[42]
	Apical resection	Lactate metabolism; redox homeostasis	CM-specific *Ldha* KO/OE	KO: reduced CM proliferation and impaired repair; OE: enhanced repair/protection	Genetic manipulation	4	[98]
Mouse (Adult)	Chronic hypoxia after MI (7% O₂; 1w post-MI)	Oxygen tension; ROS–metabolic programming	Environmental hypoxia treatment	Reduced fibrosis; increased new myocardium of CM origin (lineage tracing); functional recovery	Reproducible physiological intervention	4	[99]
	Post-MI SDH inhibition (malonate)	Mitochondrial TCA cycle	Malonate mediated SDH inhibition	CM proliferation; angiogenesis; reduced fibrosis and improved repair	Pharmacologic and metabolomics evidence	4	[100]
	I/R or MI models	Lipid metabolism; FAO–epigenetic coupling	Genetic deletion or pharmacologic inhibition of Cpt1b	CM cell-cycle re-entry; reduced fibrosis; functional recovery	Genetic and pharmacologic metabolic- epigenetic axis	4	[50]
	MI models	Mitochondrial TCA cycle	Exogenous α-KG supplementation or degradation blockade	Adult CM proliferation; improved post-MI repair	Supplementation/genetic and mechanistic evidence)	4	[101]
Porcine (Neonatal)	AMI regenerative window comparison P1/P2 vs P7	Developmental metabolic transition	Regenerative window comparison	Reduced primary scar; CM cell-cycle re-entry; rapid closure of regenerative window	Reproducible cross-species evidence (large animal)	5	[102]
Porcine (Adult)	I/R model	Mitochondrial TCA cycle	Intracoronary or systemic malonate administration	Primarily acute cardioprotection	Large-animal feasibility; physiological relevance and safety signal	5	[103]
Human	Cyanotic CHD (Tetralogy of Fallot)	Hypoxia-glycolysis axis	Clinical association study (natural experiment)	Enhanced cell-cycle markers and correlate with arterial O₂ saturation	Clinical evidence	5	[97]
Human	hPSC cardiac organoids/EHT	Metabolic transition	Culture media with defined substrate composition	improved DDR; CM cell-cycle arrest	Human causal evidence (in vitro)	5	[104]
Human	hPSC-cardiac organoid drug screening	Mevalonate pathway	Pharmacologic screen and pathway inhibition (causal)	CM cell-cycle re-entry/proliferation readouts	Human causal evidence (in vitro)	5	[105]
Human	hiPSC-CMs maturation model	Hypoxia-glycolysis axis	Inhibition of HIF1α or LDHA	State/maturation changes; CM cell cycle re-entry	Human mechanistic evidence (in vitro)	5	[106]

*TRL* Technology Readiness Level *DDR* DNA damage response

#### Glucose metabolism

Substantial evidence suggests that increased glucose metabolism (particularly increased glycolysis) plays an active role in promoting cardiac regeneration. Glucose transporter 1 (GLUT1), which is the predominant glucose transporter isoform observed in cardiomyocytes, serves as the primary mediator of cellular glucose and is essential for glycolysis [94, 107, 108]. By facilitating the transport of extracellular glucose into cardiomyocytes, GLUT1 supplies the fundamental substrate required for glycolysis. Notably, GLUT1 expression is induced under hypoxic or ischemic conditions to maintain glycolysis and ensure an adequate energy supply. GLUT1 activity directly governs glycolytic flux, thereby stimulating the production of energy carriers, including ATP and NADPH. Previous studies have consistently demonstrated that GLUT1 overexpression enhances glycolytic flux in cardiomyocytes, which concomitantly increases ATP and NADPH production [109]. The importance of GLUT1 in glycolysis has been reinforced in highly regenerative models. In zebrafish and neonatal mice, elevated GLUT1 expression is positively correlated with glycolytic activity, highlighting its contribution to the metabolic plasticity of cardiomyocytes under hypoxic stress [110, 111]. In contrast, the limited regenerative capacity observed in the adult mammalian myocardium may be associated with decreased GLUT1 expression. Similarly, the genetic ablation of GLUT1 weakens glucose uptake and glycolysis in cardiomyocytes in a transverse aortic constriction (TAC) model [112]. Notably, GLUT1 overexpression potentiates the regenerative response of neonatal mice after cryoinjury via increased accumulation of glucose metabolites [94]. Collectively, these findings establish the notion that increased GLUT1 expression facilitates cardiomyocyte proliferation and cardiac regeneration by augmenting glycolytic flux [94].

Following uptake into cardiomyocytes, glucose undergoes glycolysis to produce ATP and essential metabolic intermediates, which collectively sustain cardiomyocyte growth, development and function. Furthermore, numerous studies have demonstrated that key glycolytic enzymes play critical roles in regulating cardiac regeneration capacity. As a critical enzyme functioning in glucose metabolism, hexokinase (HK) serves as the initial rate-limiting enzyme and directly governs the efficiency of glucose entry into metabolic processes. Among the four distinct HK isoforms (HK-1, HK-2, HK-3, and HK-4), HK-1 and HK-2 are predominately expressed in cardiac tissue and regulate myocardial glucose metabolism. Specifically, HK-2 mitigates cardiac hypertrophy by increasing pentose phosphate pathway (PPP) flux to reduce ROS accumulation [113]. Reduced HK-2 levels lead to altered cardiac remodeling following ischemia/reperfusion (I/R) injury through increased apoptosis, fibrosis, and impaired angiogenesis [114]. However, the potential role of HKs in cardiac regeneration remains to be elucidated.

Downstream in the glycolytic pathway, phosphofructokinase (PFK), which is the core rate-limiting enzyme of glycolysis, catalyzes the conversion of fructose-6-phosphate (F-6-P) to fructose-1,6-bisphosphate (F-1,6-BP). Its activity is allosterically regulated by metabolites such as ATP, citrate, and AMP/ADP. A previous study demonstrated that PFK-2 enhances the contractility of hypoxic cardiomyocytes in mice, thereby indicating its role in modulating metabolism while maintaining cardiomyocyte function [115].

Pyruvate kinase M2 (PKM2) catalyzes the final glycolytic step, which transforms phosphoenolpyruvic acid (PEP) to pyruvate [43]. During the cardiac regeneration stage, increased PKM2 activity promotes a glycolysis-dominant metabolic pattern to meet proliferative demands. In addition to its metabolic function, PKM2 also acts as a transcriptional coregulator; specifically, nucleus-localized PKM2 interacts with β-catenin and HIF-1α to activate proliferation-associated genes, including *Ccnd1* and *Vegfa*, thereby directly promoting cardiomyocyte cell cycle re-entry [116]. In ischemic preconditioning (IPC), the nuclear translocation of PKM2 alleviates myocardial injury by stabilizing HIF-1α and inducing the expression of downstream protective genes such as *Hmox1* and *Pdk1* [117]. Additionally, PKM2 facilitates metabolic redirection by diverting glucose-6-phosphate (G-6-P) into biosynthetic pathways, thus promoting cardiomyocyte proliferation. Under physiological conditions, the PPP capacity in cardiac tissue is constrained by the inhibitory effect of NADPH on G-6-P dehydrogenase (G-6-PD) activity. However, PKM2 enhances G-6-PD activity, thereby promoting PPP flux and mitigating oxidative stress in cardiomyocytes. An increase in the activity of the PPP leads to increased ribonucleotide synthesis and NADPH production, which are essential for DNA synthesis and cardiomyocyte division [118]. PKM2 plays a crucial role in cardiac protection, proliferation, and regeneration by integrating metabolic and transcriptional regulatory networks. Moreover, it functions as both a metabolic enzyme and a transcriptional coactivator, thereby providing multidimensional therapeutic targets for the treatment of cardiac diseases [43].

Exposure to severe systemic hypoxemia can bypass the temporal restriction of cardiomyocyte proliferation and effectively reinitiate the regenerative potential of mature cardiomyocytes [99]. This reactivation process is consistently associated with a shift in metabolic reprogramming from oxidative phosphorylation to glycolytic metabolism. Notably, lactate (which serves as the terminal product of glycolysis) plays regulatory roles in myocardial regeneration that extend beyond its classical function as an oxidative energy source. Lactate dehydrogenase A (LDHA), which is a key enzyme involved in anaerobic glycolysis, catalyzes pyruvate-to-lactate conversion. Moreover, it is highly expressed in neonatal mouse hearts; however, this expression decreases during subsequent cardiac development. Functional studies have revealed that cardiomyocyte-specific LDHA knockout suppresses cardiomyocyte proliferation, thus leading to impaired cardiac function and reduced survival rates in a neonatal apical resection model [98]. Conversely, cardiomyocyte-specific LDHA overexpression promotes heart repair after myocardial infarction [119]. Furthermore, LDHA reduces the level of intracellular succinyl-CoA (suc-CoA) to inhibit the suc-CoA-dependent ubiquitination of Txnrd1, thereby increasing Txnrd1 protein levels, which subsequently promotes ROS reduction and cardiomyocyte proliferation [98]. Lactate is not only the end product of anaerobic glycolysis of glucose but also an important metabolic signaling molecule. Furthermore, it plays multidimensional roles in cardiac regeneration and cardiomyocyte proliferation by regulating energy metabolism, redox homeostasis, signal transduction and epigenetic modifications [120]. The embryonic heart produces abundant amounts of lactate via active glycolysis; however, metabolic reprogramming after birth leads to a marked decrease in lactate in the neonatal heart [121]. Lactate influences the regenerative microenvironment driven by LDHA-mediated lactate production, which induces the M1-to-M2 macrophage transition, inhibits inflammation and creates a pro-regenerative microenvironment [122, 123]. During cardiac ischemia, lactate sustains glycolytic flux to generate ATP and mitigate both mitochondrial oxidative stress and cardiomyocyte apoptosis [124]. Furthermore, lactate-mediated protein lactylation induces metabolic reprogramming in cardiomyocytes, thereby shifting energy metabolism toward a proliferative-permissive state. A study employing 4D label-free proteomics and lysine lactylation (Kla) omics analyses in mouse hearts revealed a positive correlation between the expression of glycolytic proteins (such as ENO1 and PKM) and lysine lactylation levels during postnatal cardiac development, whereas a negative correlation was observed with fatty acid oxidation-related proteins (such as ACADL and ACAT1) [124]. Additionally, lactate directly regulates gene expression via the histone lactylation-mediated transcriptional reprogramming of protective genes. At the early stage of MI, histone H3K18 lactylation in cardiac-infiltrating macrophages facilitates the transcription of *Lrg1*, *Vegfa* and *Il10*, thus enhancing their multiple functions in the anti-inflammatory and pro-angiogenic activities of macrophages. Research has demonstrated that exogenous lactate supplementation can reduce myocardial infarction size by approximately 25% [125].

In addition to glycolytic enzymes, other key enzymes involved in aerobic glucose oxidation critically regulate cardiac regeneration. A prime example of this process includes pyruvate dehydrogenase kinase (PDK), which serves as the essential regulatory enzyme of the pyruvate dehydrogenase complex (PDHc) and modulates PDHc activity via phosphorylation, along with controlling the conversion of pyruvate to acetyl-CoA. This enzymatic regulatory mechanism fundamentally governs the metabolic flux direction; specifically, the PDK-mediated inhibition of PDHc reduces acetyl-CoA generation and promotes glycolysis (anaerobic metabolism), whereas PDHc activation enhances the TCA cycle (aerobic metabolism). Crucially, PDK dynamically responds to physiological conditions. Specifically, its activity is regulated by metabolic status (including hyperglycemia and fatty acid levels) and hormonal signals (such as insulin) [126, 127]. For example, elevated PDK activity during hyperglycemic conditions can inhibit PDHc to reduce glucose oxidation and promote fatty acid metabolism. In cancer cells, the hyperactivation of PDK enhances glycolytic flux even in aerobic environments, which is a phenomenon known as the Warburg effect [128]. Emerging evidence suggests that metabolic reprogramming may play a critical role in cardiac regeneration. However, the limited regenerative capacity of adult mammalian hearts is partially attributable to metabolic inflexibility that is predominantly driven by fatty acid oxidation and the reduced activity of key glycolytic enzymes [50]. Research has demonstrated that PDK3 overexpression promotes cardiomyocyte proliferation in zebrafish models by augmenting glycolytic flux, whereas PDK4 exhibits the opposite effect [51]. Specifically, the knockout of PDK4 or its inhibitors significantly enhances the regenerative capacity of cardiomyocytes in adult mice [95, 129]. Furthermore, PDK modulates mitochondrial oxidative phosphorylation (OXPHOS) by regulating PDHc activity. The metabolic pattern shift from OXPHOS to glycolysis represents a critical reprogramming step in cardiac regeneration [129]. Importantly, PDK inhibition confers cardiac protection by attenuating oxidative stress via increased NADH generation and the activation of antioxidant pathways, thereby preventing cardiomyocyte damage.

In summary, these studies demonstrate that glucose metabolism serves as a critical metabolic pathway that regulates cardiomyocyte proliferation and cardiac regeneration. The targeting of key regulatory factors of cardiomyocyte glycolysis demonstrates significant promise as an effective therapeutic strategy for ischemic heart disease and heart failure.

#### Lipid metabolism

Postnatally, cardiomyocytes undergo a metabolic shift from glycolysis to FAO, which is accompanied by epigenetic remodeling and cell cycle withdrawal. This process drives the remodeling of mitochondrial homeostasis, thus ultimately leading to cardiomyocyte maturation and entry into a terminally differentiated state, which results in the loss of regenerative capacity [95]. The key FAO-dependent enzyme known as carnitine palmitoyl transferase 1b (Cpt1b) plays a central role in this process [50]. Cpt1b is primarily responsible for the β-oxidation of fatty acids, facilitating their transport from the cytoplasm into the mitochondrial inner membrane of cardiomyocytes for further decomposition to generate energy [130]. Research has demonstrated that etomoxir-mediated Cpt1b inhibition reverses cardiomyocyte metabolic maturation and restores proliferation in rodent and mice models. Cpt1b deletion in adult mouse hearts triggers cell cycle reactivation and ventricular hypertrophy. In the I/R injury model, *Cpt1b* KO mice exhibit a lower degree of fibrosis and fully restored contractile function. Moreover, the suppression of FAO metabolism alters the TCA cycle and enhances isocitrate dehydrogenase (IDH) activity, thereby resulting in a 20-fold increase in α-ketoglutarate (α-KG) levels [50]. Moreover, α-KG serves not only as an essential mediator but also as an essential cofactor for histone demethylases (KDMs). Mechanistically, α-KG activates the lysine demethylase known as KDM5 to specifically remove H3K4me3 marks (which represent hallmark modifications of active transcriptional promoters), thereby repressing the expression of cardiomyocyte maturation genes such as *Tnni3* and *Myocd* and promoting their reversion to the dedifferentiated state [101]. Another study revealed that α-ketoglutarate (α-KG) is one of the most significantly downregulated metabolites during postnatal cardiac development. Subsequent research demonstrated that exogenous α-KG supplementation promotes adult cardiomyocyte proliferation via JMJD3 (KDM6b)-dependent demethylation to reduce the inhibitory histone mark H3K27me3 on cell cycle genes (such as *Ccna2*, *Ccnb1*, and *Cdk1/4*, among others), thus consequently enhancing cardiac regeneration after MI. Furthermore, the genetic ablation of *Ogdh* to inhibit α-KG degradation similarly enhances cardiomyocyte proliferation, thereby confirming its pro-regenerative effect. Fatty acid metabolic reprogramming reshapes the chromatin landscape via the α-KG-KDM5/KDM6b axis, thus alleviating the transcriptional repression of maturation genes and establishing an epigenetic foundation for cell cycle re-entry [101]. This mechanism dynamically couple energy metabolism with gene regulation, which creates multidimensional regulatory points for cardiac regeneration.

Another lipid metabolite that can effectively promote myocardial regeneration is arachidonic acid (AA). As an ω−6 polyunsaturated fatty acid, AA is extensively distributed in membrane phospholipids and generates bioactive metabolites through the catalysis of cyclooxygenase (COX), lipoxygenase (LOX), and cytochrome P450 (CYP). AA-derived metabolites play crucial roles in regulating inflammation, vascular homeostasis and cellular proliferation [131]. In response to adult cardiac injury, macrophages typically promote scar formation. Conversely, within the neonatal heart, macrophages are an essential cell type for initiating regeneration [132]. Recent research has revealed that this functional discrepancy stems from differential AA metabolism in tissue-resident macrophages between neonates and postinjury adults [42]. In neonatal hearts, macrophage-derived AA is metabolized via the cyclooxygenase-1 (COX-1) pathway to generate thromboxane A2 (TXA2). TXA2 subsequently activates its cognate receptor known as Tbxa2r on cardiomyocytes, triggers YAP/TAZ signaling and ultimately promotes the expression of downstream factors (such as Ctgf and Slc2a1). This cascade enhances both glycolytic metabolism and the cell cycle process in nascent cardiomyocytes, thus ultimately driving cardiac regeneration [42].

As a crucial cholesterol-derived steroid hormone. estradiol represents specific regulating roles in sex-specific cardiac regeneration. Comparative studies in zebrafish highlight a distinct sexual related program in heart repair [133]. Female zebrafish display a stronger regenerative capacity following cardiac injury that exhibit significantly higher expression of proliferation-related markers compared to males [134]. Various experimental models further validate this estrogen-driven regenerative advantage. In vivo zebrafish models using cryoinjury and apical resection confirm that endogenous estrogen accelerates myocardial repair. Exogenous cardiomyocyte cultures demonstrate consistent results. Mechanistically, estrogen promotes this repair process by upregulating key pro-inflammatory mediators. It specifically enhances the expression of interferon-gamma (IFN-γ) and signal transducer and activator of transcription 1 (Stat 1). This targeted upregulation amplifies the injury-induced local inflammatory response. Consequently, the remodeled immune microenvironment facilitates cardiac tissue repair and regeneration [134].

Unlike simple lipids such as fatty acids and arachidonic acid, complex lipids such as sphingomyelin also affect cardiac regeneration through developmental and proliferative pathways. Sphingolipids constitute a critical class of lipid molecules that play versatile roles in membrane architecture, signal transduction and metabolic regulation [135, 136]. Emerging evidence reveals that sphingolipid metabolic homeostasis serves as a key regulator of cardiac regenerative capacity [41]. Ceramide serves as the main precursor of sphingolipids in cardiomyocytes and is metabolized by sphingosine kinase (SphK) in the endoplasmic reticulum and mitochondria to generate sphingolipid metabolites, such as sphingosine-1-phosphate (S1P). S1P functions as an indispensable signaling molecule that modulates cellular proliferation, migration, and inflammatory responses by binding to its cognate G protein-coupled receptors (including S1PR1, S1PR2, S1PR3, S1PR4, and S1PR5). The importance of the S1P pathway has been highlighted by studies conducted across various species. In zebrafish, the SPHK2-SPNS2-S1PR2-Hippo signaling axis is essential for endoderm patterning and cardiac progenitor cell migration during development [137]. Additionally, murine studies have demonstrated that S1P signaling in endocardial, cardiomyocyte and endothelial cell populations plays indispensable roles in embryonic heart morphogenesis [138]. Moreover, in human induced pluripotent stem cells (hiPSCs), S1P promotes cell cycle initiation via increased ERK signaling [139]. Furthermore, S1P-targeted therapeutic strategies have also demonstrated efficacy in cardiac injury models. In a rat MI model, researchers employed a liposome-incorporated hydrogel containing S1P to activate the S1PR1/PI3K/Akt pathway, which could stimulate endothelial cell angiogenesis and scavenge excess ROS to ameliorate mitochondrial dysfunction [140]. When further considering the regulation of S1P production, the SphK isoforms SphK1 and SphK2 differentially regulate cardiac regeneration through distinct S1P production pathways. In neonatal hearts, nucleus-localized SphK2 generates S1P, which maintains cardiomyocyte proliferative capacity by inhibiting histone deacetylase (HDAC) activity to increase the transcriptional activation of *Erbb4*, *Mef2a *and *Mef2c *via H3K9/H3K27 acetylation. Conversely, adult hearts exhibit diminished SphK2 expression, thus resulting in reduced nuclear S1P levels and cell cycle arrest. During the maturation process, SphK1 becomes the dominant isoform and drives cardiac fibrosis through autocrine S1P-mediated activation of TGF-β-Smad2/3 signaling. The pharmacological adjustment of SphK1/2 isozyme activity represents a promising therapeutic strategy to enhance myocardial repair while limiting fibrotic scar formation, thereby enabling regeneration in infarcted adult hearts. In neonatal mouse apical resection model, another study demonstrated that S1PR1 overexpression promotes cardiomyocyte proliferation by potentiating AKT/mTORC1/Cyclin D1 signaling. Concurrently, S1PR1 activation suppresses apoptosis via the AKT/BCL2 axis, ultimately ameliorating post-injury cardiac function [141].

Building on this metabolic shift, ketone body metabolism emerges as a closely related pathway that further integrates lipid utilization with postnatal cardiac maturation and regenerative decline. During the postnatal period, ketone metabolism appears to function primarily as a component of a metabolic maturation brake that constrains cardiomyocyte proliferation. Tanaka et al. reported that increased fatty acid availability activates PPARδ signaling in neonatal mouse cardiomyocytes, leading to the upregulation of *Pdk4* and *Hmgcs2* in parallel with a decline in cell-cycle activity [142]. Mechanistically, PDK4 suppresses pyruvate dehydrogenase activity and limits glucose oxidation, whereas HMGCS2 enhances mitochondrial ketone body synthesis [142]. Within this context, elevated ketone-generating capacity is best interpreted as a marker of fatty-acid–driven metabolic maturation rather than a pro-regenerative signal. Consistent with this view, induction of the PPARδ–PDK4–HMGCS2 axis coincides with cardiomyocyte cell-cycle exit and the closure of the neonatal regenerative window.

Conversely, ketone metabolism assumes a distinct and permissive role in in the adult heart under injury or reprogramming conditions. Cheng et al. reported that cardiomyocyte specific induction of a dedifferentiation program is accompanied by marked metabolic remodeling, in which HMGCS2-mediated ketone body production is required for an effective injury response [143]. In injured microenvironment, ketone generation supports cellular state transitions associated with partial dedifferentiation and re-entry into the cell cycle, rather than reflecting terminal metabolic maturation. Zhang et al. reported that ginsenoside Rd promotes cardiac regeneration through PPARG/HMGCS2-driven ketone body metabolic reprogramming in I/R models [144]. Together, these findings indicate that the impact of ketone metabolism on cardiac regeneration is highly context dependent: it reinforces metabolic maturation and proliferation arrest during postnatal development, yet may facilitate metabolic adaptation and regenerative competence in the injured adult myocardium.

Fatty acid oxidation occurs not only in mitochondria but also in peroxisomes, where it plays a critical role in regulating redox balance and influencing cardiac repair following injury. Peroxisomes process very long-chain and complex lipids through FAO and ether lipid biosynthesis, mitigating lipotoxic stress and maintaining cellular redox homeostasis. Recent studies in mice have shown that conditional deletion of the peroxisomal biogenesis factor PEX3 in cardiomyocytes disrupts redox balance, impairs cell proliferation, and leads to cardiac developmental defects. In models of apex resection or myocardial infarction, *Pex3*-deficient hearts exhibit reduced cardiomyocyte cell-cycle activity, increased scar tissue formation, and diminished functional recovery. Restoration of phosphatidylethanolamine levels rescues cardiac regeneration via ITGB3-mediated AKT-GSK3β signaling [145]. These findings highlight that peroxisomal lipid metabolism and FAO are essential not only for detoxification but also for maintaining redox balance and promoting regenerative signaling in injured hearts [145].

In summary, postnatal lipid metabolic reprogramming in cardiomyocytes represents an instrumental mechanism underlying the loss of cardiac regenerative capacity. The dynamic interplay between lipid homeostasis and epigenetic remodeling forms a core regulatory network that governs regeneration.

#### Amino acid metabolism

Amino acids constitute the primary structural units of biologically functional proteins. In addition to their role in protein synthesis, amino acids participate in various metabolic processes and play regulatory roles in physiological functions. These essential molecules are indispensable for normal cellular growth, differentiation, and maintenance of cellular architecture and energy homeostasis. Critically, novel evidence highlights the role of amino acid metabolism in sustaining cell proliferation, energy provision and antioxidant defense mechanisms. Metabolomic profiling of murine cardiac tissues has revealed that the levels of most amino acids are increased during early postnatal stages but decrease during adolescence [146]. Moreover, the levels of specific amino acids, including histidine, cystine, leucine, isoleucine, valine, lysine, and tyrosine, are significantly increased during cardiomyocyte dedifferentiation (CMDD) [147]. These findings underscore the role of amino acids as essential biomolecules in cardiac development and regeneration.

Among amino acids, glutamine serves both as a substrate for protein synthesis and as a critical modulator of energy metabolism and epigenetic regulation, orchestrating myocardial proliferation and tissue repair. The metabolic mode of glutamine is primarily governed by glutaminase (GLS1)-mediated conversion to glutamate, which is subsequently transformed into α-KG for entry into the TCA cycle. This pathway simultaneously provides precursors for nucleotide and lipid biosynthesis, maintains redox homeostasis and regulates signaling networks to meet regenerative demands. In highly regenerative models such as neonatal mice and zebrafish, cardiac injury triggers an increase in glutamine uptake and metabolism. Elevated glutamine levels in cardiomyocytes activate the amino acid-driven mTOR signaling pathway and promote mitochondrial maturation to increase cardiomyocyte proliferation [148]. Sun et al. reported that exogenous glutamine supplementation can prevent cardiomyocyte apoptosis, facilitate cardiac repair, and ameliorate azithromycin-induced cardiac dysfunction [149, 150]. The therapeutic implications are attributed to glutaminolysis-derived α-KG and its ability to generate ATP and NADPH. In addition, glutamine promotes macrophage polarization, inhibits proinflammatory mediators such as TNF-α and upregulates reparative factors such as IL-10 to create permissive conditions for regeneration [151].

In addition to essential amino acids, accumulated nonessential amino acids and their metabolites impact myocardial regeneration, as exemplified by the functions of asparagine. Asparagine is synthesized from aspartate and glutamine via asparagine synthetase (ASNS). RNA sequencing and comprehensive analysis have identified the asparagine synthetase (ASNS) gene as a unique molecular marker gene that is closely associated with CMDD and is essential for increasing asparagine and for the differential flux of other amino acids [152]. Functionally, ASNS deficiency not only reduces the cardiomyocyte asparagine content but also suppresses cell cycle re-entry and survival during CMDD. Recent studies have revealed that the depletion of asparagine via L-asparaginase treatment leads to inhibited expression of cell cycle markers (such as Ki67, pH3, and Aurora kinase B) within proliferating cardiomyocytes [152]. Furthermore, elevated asparagine levels induce the accumulation of glutamine synthetase (GLUL) in cardiomyocytes, which promotes glutamine synthesis and activates mTORC1 signaling targets (including S6K1 and 4E-BP1) to drive cardiac regeneration [153, 154].

These findings underscore the potential role of amino acid metabolic networks in cardiac regeneration, which establishes a molecular foundation through energetic metabolism, signal transduction and cell cycle regulation. In addition to supplying energy, amino acids promote cardiac regeneration through regulatory effects that are independent of protein synthesis.

#### Mitochondrial remodeling and biogenesis in cardiac repair and regeneration

Traditionally viewed as a passive byproduct of cardiomyocyte maturation, mitochondrial biogenesis is now recognized as a dynamic determinant of regenerative competence [155]. Successful myocardial repair necessitates more than the mere suppression of oxidative metabolism; it requires a precise reconfiguration of the mitochondrial network to establish a metabolically permissive environment for the cell cycle [156, 157]. By orchestrating the supply of essential building blocks and re-balancing the cellular redox landscape, these pathways enable cardiomyocytes to transition from a terminally differentiated oxidative state toward a reparative, proliferation-competent phenotype.

Mitochondrial biogenesis is increasingly recognized as an active determinant of regenerative competence rather than a passive consequence of CM maturation. Emerging evidence indicates that successful cardiac regeneration requires a precisely regulated re-establishment of mitochondrial capacity instead of a simple suppression of oxidative metabolism [158]. Recent studies demonstrate that the induction of mitochondrial biogenesis by CLIPPER-LPIN1 signaling can directly promote cardiomyocyte proliferation and functional recovery after injury [159]. These findings support the concept that regeneration depends on a metabolically permissive state in which mitochondrial content and functionality are tuned to sustain proliferative growth. Within this framework, metabolite-sensitive regulatory networks govern mitochondrial biogenesis rather than energy demand alone. Among these networks, the one-carbon (1C) folate pathway and the NAD^+^-sirtuin axis have emerged as key upstream modulators linking biosynthetic capacity to cardiomyocyte plasticity.

The mitochondrial 1C/folate pathway serves as a central biosynthetic hub supplying nucleotide precursors, redox equivalents, and methyl donors required for cell-cycle progression. Genetic disruption of mitochondrial 1 C metabolism, such as the loss of SHMT2, results in profound cardiac developmental defects and compromised mitochondrial integrity, which underscores its fundamental role in myocardial growth [160]. The necessity of this pathway is further evidenced fetal mice models that maternal folate availability orchestrates the embryonic CM proliferation, which suggests that 1 C metabolism constitutes a core biosynthetic engine for cardiac growth [161]. Beyond the role as a passive source of raw materials, 1 C metabolism functions as a metabolic rheostat that can be reprogrammed under pathological stress. For instance, the mitochondrial enzyme MTHFD1L acts as a metabolic-to-cell-cycle coupler that can divert metabolic flux toward endoreplication and polyploidization, thereby increasing the structural barriers to successful regeneration [162]. Although direct regeneration-specific evidence remains limited, the requirement for sustained nucleotide biosynthesis and redox balance during cardiomyocyte cell-cycle re-entry positions mitochondrial 1 C metabolism as a permissive program enabling injury-induced proliferative reprogramming.

In parallel, the NAD⁺–sirtuin network operates as a metabolic switch that decodes cellular energy status to orchestrate mitochondrial biogenesis. Sirtuins function as critical homeostatic regulators by integrating mitochondrial biogenesis with antioxidant defenses and quality control mechanisms [163]. Specifically, SIRT3 coordinates mitochondrial biogenic programs with oxidative stress responses to maintain the mitochondrial integrity required for survival during metabolic shifts. Beyond stress adaptation, the sirtuin family directly influences the molecular decision-making of the cell cycle by modulating the acetylation status of key regenerative effectors. A primary example of this metabolic-epigenetic coupling is the NAD⁺-dependent regulation of YAP signaling [77]. Conversely, the loss of SIRT4 removes a metabolic brake on cardiomyocyte proliferation and effectively extends the neonatal regenerative window [164]. Furthermore, SIRT1 promotes proliferative competence following injury by deacetylating key cell-cycle regulators, which facilitates the necessary transition from a terminal oxidative state back to a cycling phenotype [165]. These findings collectively suggest that NAD⁺-dependent deacetylation programs serve as a permissive gateway, adjusting mitochondrial capacity to support the metabolic demands of cardiomyocyte regeneration [165]. Collectively, these metabolite-coupled networks converge to define a mitochondrial state compatible with regeneration [166]. The 1C–folate system provides the anabolic and redox infrastructure necessary for proliferative growth, while the NAD⁺–sirtuin axis modulates mitochondrial biogenic capacity in response to metabolic cues [167]. Through the coordinated regulation of mitochondrial biogenesis, these pathways may establish a permissive metabolic environment that enables cardiomyocytes to shift from a terminally differentiated oxidative state toward a reparative and proliferation-competent phenotype [168].

Beyond the expansion of mitochondrial mass, the regenerative potential of CM is fundamentally governed by the redox/cofactor economics, where the balance of NADH/NADPH partitioning serves as a metabolic sensor of proliferative capacity. Mitochondrial biogenesis does not merely increase energy production but fundamentally reconfigures the energy landscape to favor biosynthetic flux over oxidative degradation [95]. This transition necessitates a strategic reallocation of metabolic resources, moving away from oxygen-intensive oxidative phosphorylation (OXPHOS) toward pathways that support the anabolic requirements of the cell cycle [93, 156].

Central to this metabolic reconfiguration is the requirement for a robust NADPH-dependent reductive capacity, which acts as the fuel for both de novo biosynthesis and the neutralization of ROS [169]. The initiation of cardiac regeneration depends heavily on the redirection of glucose carbon flux toward the pentose phosphate pathway (PPP) to bolster the NADPH pool [170]. As demonstrated by Magadum et al., the over-expression of PKM2 facilitates this shift by enhance the production of G-6-PD, thereby increasing NADPH production and nucleotide supply [43]. This metabolic maneuver effectively attenuates oxidative DNA damage and provides the necessary metabolic entry for CM cell-cycle re-entry. The critical nature of this NADPH budget is further highlighted by the postnatal closure of the regenerative window. Saito et al. reported that the transition from neonatal to adult life is marked by a metabolic switch from purine synthesis to purine catabolism, a process that depletes the PPP flux and leads to a precipitous decline in NADPH reserves [171]. By inhibiting xanthine metabolism, it is possible to maintain the NADPH budget and successfully extend the window of regenerative potential.

In parallel with the demand for NADPH, the management of the NADH-driven oxidative load serves as a primary constraint on cardiomyocyte plasticity. While OXPHOS efficiently sustains the high ATP demands of the adult heart, the resulting mitochondrial ROS production acts as a molecular brake on cytokinesis. Strategic disinvestment from this oxidative budget has proven to be a potent regenerative strategy. For example, the competitive inhibition of succinate dehydrogenase (SDH) by malonate reduces mitochondrial ROS output and alleviates the DNA damage response (DDR) [100]. This metabolic deceleration lowers the threshold for cell-cycle re-entry, a logic further supported by the regenerative effects of chronic hypoxia, which forces a shift away from NADH-intensive mitochondrial respiration toward a more "regeneration-friendly" redox state.

At the intersection of these cofactor pools lies the nicotinamide nucleotide transhydrogenase (NNT), a mitochondrial enzyme that functions as a critical interface for metabolic exchange [172]. By utilizing the mitochondrial proton gradient to transfer reductive equivalents from NADH to NADPH, NNT facilitates the partitioning of reducing power toward antioxidant regeneration and biosynthetic pathways. Through the coordinated regulation of these redox exchangers and the strategic partitioning of cofactor fluxes, cardiomyocytes establish a permissive environment that enables the transition from a terminally differentiated oxidative state toward a proliferation-competent phenotype [173].

The coordinated regulation of mitochondrial biogenesis and redox economics provides a transformative therapeutic blueprint for unlocking the heart's endogenous regenerative potential. By targeting specific metabolic checkpoints, such as the NAD^+^—sirtuin axis and the NNT-driven redox exchange, clinicians facilitate the pharmacological avenue to induce a pro-proliferative type in terminally differentiated CMs. This strategy shifts the focus of cardiac repair from passive scarring toward active metabolic rejuvenation, leveraging reductive power to drive biosynthesis while neutralizing oxidative DNA damage. Ultimately, re-balancing the NADH/NADPH balance serves as a molecular switch to transition the failing heart from a state of terminal arrest to pro-proliferation restoration. These pathways thus represent a scalable framework for the next generation of regenerative medicine interventions.

### Metabolic remodeling in clinical cardiac therapies: implications and gaps

Cardiac injury in adult mammals is consistently accompanied by metabolic remodeling. Beyond changes in ATP production, metabolic alterations affect mitochondrial function, redox homeostasis and the availability of metabolites that regulate biosynthesis and chromatin modification. Emerging evidence indicates that such disease-associated metabolic states are closely linked to cardiomyocyte cell-cycle arrest and loss of regenerative capacity. Thus, the metabolic context of the injured heart is increasingly recognized as a determinant of intrinsic regenerative competence (Table 2).

**Table 2:** The pre-clinical landscape of cardiac regeneration targets: efficacy, consistency, and current challenges

Molecules/pathway	Objects	Effects	Article numbers	Consistency	Negative/neutral reports	Challenges
GLUT1/Glucose uptake	Neonatal mouse; zebrafish	Enhanced glycolytic flux promotes CM proliferation	2–3	Moderate	Limited	Limited adult mammalian translation; effect sizes are inconsistently reported
SDH (succinate dehydrogenase)	Neonatal/adult mouse	Decreased metabolites in the TCA cycle such as α-KG, increased succinate and metabolites of glucose metabolism	1	Low	Limited	Limited adult mammalian translation
PKM2	Neonatal/adult mouse; porcine models	PKM2 activation supports anabolic metabolism and cell-cycle re-entry	2–3	Moderate	Limited	Systemic PKM2 activation may entail oncogenic risk due to nuclear PKM2
LDHA	Neonatal mouse; lactate-enriched regeneration models	Elevated lactate production supports CM proliferation	2–3	Moderate	Limited	The role of lactate acting as a signal molecule or metabolic by-product remains debated
PDK3	Rat neonatal cardiomyocytes; zebrafish	Regulation of pyruvate oxidation modulates CM plasticity	2–3	Moderate	Limited	Stage-specific requirements and adult mammalian safety windows remain undefined
Cpt1b/FAO suppression	Neonatal/adult mouse; zebrafish	FAO suppression enables adult CM regenerative responses	2–3	Moderate	*Cpt1b* ablation suppresses CM proliferation in zebrafish (39,590,187)	Inconsistent regenerative effects of Cpt1b ablation
HMGCS2	hiPSC derived CM and Microarray analysis	Regulation of ketogenesis allowing cell dedifferentiation and proliferation after injury	2	Moderate	HMGCS2 induced ketogenesis inhibit the CM development from P8	Inconsistent regenerative effects of *Hmgcs2* ablation
AA/TXA2 – TP receptor	Macrophage–cardiomyocyte crosstalk models	Thromboxane signal proposed to stimulate CM proliferation	1	Low	Limited	Reproducibility and separation of vascular versus CM-intrinsic effects remain major gaps
S1P/S1PR1	Neonatal/adult mouse	S1PR1 activation promotes regenerative signal via AKT/mTORC1	2	Moderate	S1P-S1PR3 signaling promotes spontaneous ventricular fibrosis.	Strong cell-type specificity; developmental versus regenerative roles remain difficult to clarify interventions
Hippo–YAP	Neonatal/adult mouse; rodents and swine; porcine models	YAP activation reopens CM plasticity and glycolytic programs	≥ 5	High	Limited	Whether metabolic reprogramming is upstream or downstream of YAP activation remains unresolved
PI3K–AKT–mTOR	Neonatal/adult mouse; zebrafish	Anabolic signal supports growth and regenerative programs	≥ 5	Moderate	Limited	Difficult to distinguish regeneration from hypertrophy; standardized effect-size reporting is lacking

When exposed to ischemia or overloading pressure, the heart undergoes remodeling of mitochondrial function, substrate preference and metabolic flux rather than a simple switch from FAO to glucose use. Recent researches in animal models and human samples indicate that the pattern and severity of this remodeling closely track the underlying disease etiology and stage of heart failure (HF) [174, 175]. Experimental studies have indicated that during acute I/R injury, there is a compensatory increase in glycolytic flux to maintain ATP levels. However, limited oxygen availability restricts glucose oxidation. This metabolic imbalance leads to intracellular acidosis and calcium overload, thereby exacerbating reperfusion injury and contractile dysfunction [176]. In chronically failing hearts, transcriptomic and metabolomic profiling revealed a distinct transition from compensated to decompensated states. This shift is characterized by downregulated FAO, a relative increase of glycolysis despite impaired glucose oxidation, and altered amino acid catabolism. Collectively, this progressive loss of metabolic flexibility parallels the decline in phosphocreatine/ATP ratios and systolic function [175, 177].

Importantly, these metabolic shifts are not merely passive consequences but also active drivers of ventricular remodeling and failure. Using quantitative mitochondrial proteomics, by employing ^13^C6–lysine tracer flux and metabolomics in mouse models of transverse aortic constriction and myocardial infarction, Aubert et al. demonstrated that the hypertrophied and failing heart suppresses FAO-related enzymes and upregulates β-hydroxybutyrate dehydrogenase 1, resulting in a marked increase in ketone-body oxidation, serving as a primary source for oxidative ATP production [178]. Complementary.

metabolomic profiling of severely failing human hearts revealed that advanced heart failure is associated with disruption of intramyocardial lipid metabolism, depletion of intermediates in the TCA circle, and increased myocardial ketone utilization with the induction of ketone-oxidation enzymes [179]. Recent studies utilizing arteriovenous metabolomics and tissue profiling have confirmed a profound metabolic reconfiguration in end-stage human cardiomyopathy. Specifically, the failing heart exhibits increased utilization of ketone bodies and its substrates, accompanied by a simultaneous decline of long-chain fatty acids and branched-chain amino acids (BCAAs) [180]. At the amino-acid level, impaired BCAA catabolism and the accumulation of branched-chain ketoacids (BCKAs) have been shown to promote pathological hypertrophy and dysfunction in experimental models, whereas the restoration of this pathway ameliorates remodeling [181]. Subsequent analyses of large prospective cohorts have established elevated circulating BCAAs as independent predictors of incident cardiovascular events and heart failure. These findings highlight the profound prognostic significance of amino acid metabolic reprogramming in cardiovascular disease [182, 183].

In contrast, therapeutic metabolic reprogramming seeks to temporarily revive a developmental anabolic state, coupling active biosynthesis with chromatin relaxation to physically enable cardiomyocyte division. Accumulating evidence indicates that specific metabolites and enzymes serve as critical regulatory hubs within the cardiac regenerative framework. This recognition has positioned metabolic pathways as therapeutic points in ischemic heart disease and heart failure. Therefore, the focus of contemporary therapeutics lies in securing cardiac energetics through the targeted optimization of substrate preference, mitochondrial function, and redox homeostasis.

Within current guideline-directed HF therapy, sodium glucose cotransporter 2 inhibitors (SGLT2i) are a principal example. Large-scale trials such as DAPA-HF and EMPEROR-Reduced showed that dapagliflozin and empagliflozin reduce the composite of cardiovascular death and HF hospitalization across ejection-fraction ranges and irrespective of diabetes status [184, 185]. Mechanistic work indicates that SGLT2 inhibition induces a chronic, mild ketogenic state, shifts fuel use toward ketone bodies and fatty acids, improves mitochondrial function and reduces oxidative stress, thereby improving cardiac energetics and remodeling [186, 187]. Emerging as another profound breakthrough in non-traditional cardiovascular medicine, intravenous iron supplementation has become a pivotal intervention in heart failure. The efficacy of this strategy, extensively validated in the FAIR-HF, CONFIRM-HF, and EFFECT-HF trials, includes consistent improvements in functional capacity, health status, and hospitalization rates among iron-deficient patients. These robust clinical outcomes reinforce the concept that correcting iron-dependent deficits in mitochondrial function and oxygen transport represents a core metabolic strategy rather than a secondary adjunct to standard care [188–190]. The primary endpoints of these therapies are functional improvement and attenuation of adverse remodeling.

CPT1 inhibition represents a promising metabolic strategy for heart failure while different CPT1 inhibitors may target distinct isoforms. Oxfenicine exhibits a remarkable degree of tissue specificity toward the cardiac/muscle isoform CPT1B, supporting its relevance in heart failure models [191, 192]. By contrast, etomoxir is generally considered to target the liver isoform CPT1A, which may partly explain its systemic metabolic effects and safety concerns [193, 194]. Experimental studies showed that oxfenicine delayed ventricular decompensation and attenuated adverse remodeling in pacing-induced heart failure [191], whereas early clinical studies of etomoxir suggested functional benefit but were limited by hepatic toxicity [193]. Perhexiline which characterized as a broad-spectrum metabolic modulator, provided stronger clinical evidence in improving exercise capacity, LVEF and skeletal muscle energetics, although therapeutic drug monitoring is required [195]. Collectively, these studies suggest that CPT1 remains an attractive but complex target. Future development should prioritize isoform-selective, reversible, and stage-specific modulation rather than non-selective CPT1 blockade.

A clear conceptual distinction is required to position metabolic intervention within the framework of cardiac regeneration. Metabolic optimization is primarily directed toward improving energetic efficiency, maintaining mitochondrial homeostasis and attenuating cardiac remodeling, with functional stabilization as its principal endpoint. In contrast, regenerative metabolic reprogramming seeks to transiently reshape a development-like state characterized by enhanced biosynthetic flux and chromatin plasticity, thereby creating a metabolic microenvironment permissive for cardiomyocyte cell-cycle re-entry. The divergence between these paradigms defines the central translational gap in metabolic therapy for regeneration. Enhancement of energetic efficiency, although necessary, is not sufficient to confer regenerative metabolic competence.

Ultimately, contemporary clinical interventions converge on a unified metabolic objective. By optimizing substrate utilization, maintaining mitochondrial integrity, and restoring redox homeostasis, these strategies collectively maximize energetic efficiency and attenuate adverse ventricular remodeling.

### Epigenetic remodeling in cardiac repair and regeneration

Epigenetic reprogramming has emerged as a fundamental mechanism governing cellular plasticity and tissue repair. In the context of cardiac injury, this regulatory process orchestrates the specific gene expression profiles essential for cardiomyocyte renewal and functional recovery. This epigenetic plasticity is governed by cellular metabolism, a relationship clearly illustrated by the developmental loss of regenerative capacity in adult mice hearts. Specifically, the postnatal metabolic transition from glycolysis to fatty acid oxidation drastically alters the intracellular pool of available metabolites. Because these molecules also act as essential substrates and cofactors for chromatin-modifying enzymes, their fluctuation establishes a direct metabolism-epigenetics axis. Consequently, this biochemical interplay ultimately dictates the transcriptional programs required for effective cardiac repair.

The foundational role of epigenetic regulation underscores the involvement of specific chromatin modifiers that directly influence the gene expression programs responsible for cardiac regeneration, highlighting the complexity of these mechanisms in facilitating cellular responses to injury and repair. Acetylation-induced stabilization of Notch1 enhances cardiomyocyte regenerative activity [196], whereas Hdac2–Hopx-mediated deacetylation of Gata4 enforces an anti-proliferative state [197]. Histone methylation exerts similarly nuanced control: EZH1-mediated monomethylation at H3K27 activates growth-associated gene programs such as *Ccna2*, *Ccnb1* and *Cdk1/4* [198], and Smyd1-dependent methylation of Tribbles3 supports regenerative signaling [199]. Demethylases add an additional layer of flexibility. KDM6b removes the repressive H3K27me3 mark and KDM4d removes H3K9me3, thereby de-repressing proliferation-associated loci and facilitating cell-cycle re-entry [200, 201]. The lysine-specific demethylase LSD1 suppresses the active H3K4me2 mark at the promoter of the cell-cycle exit and neuronal differentiation gene *Cend1*, and reduced *Cend1* expression promotes cell-cycle progression during the differentiation of induced pluripotent stem cells into cardiomyocytes in vitro [202]. Further research in postnatal murine hearts demonstrated the LSD1 supports neonatal heart regeneration through suppressing CEND1 [203].

**Fig. 5:**
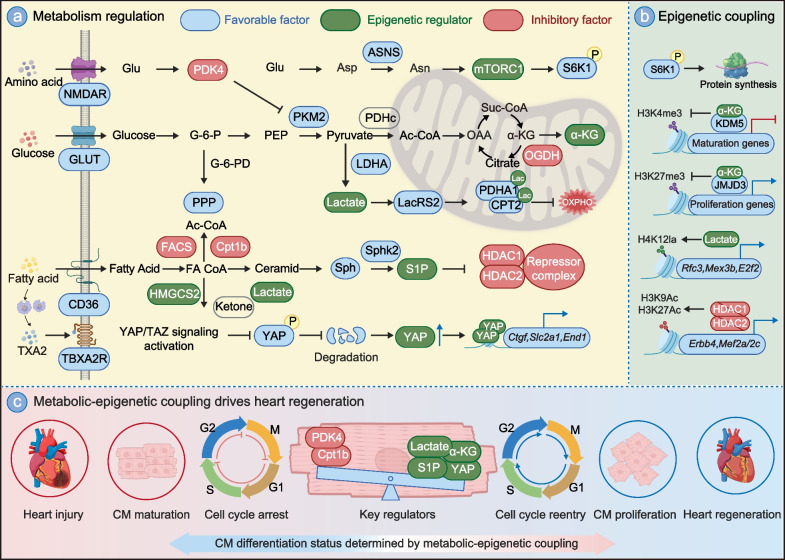
Metabolic-epigenetic-regeneration readout circle drives cardiac regeneration. **a** Metabolism regulation: Distinct metabolic pathways involving amino acids, glucose, and fatty acids are dynamically regulated within cardiomyocytes. The uptake of these extracellular substrates through specific transporters or receptors (NMDAR, GLUT, CD36) leads to the intracellular generation of key metabolic intermediates. Certain metabolites, such as α-ketoglutarate (α-KG), lactate, and sphingosine-1-phosphate (S1P), function as favorable epigenetic regulators. Conversely, factors including PDK4 and Cpt1b act as inhibitory factors. Furthermore, these metabolic cascades intersect with major signaling networks, notably facilitating YAP activation. **b** Epigenetic coupling: Key metabolites derived from the aforementioned pathways directly serve as substrates or cofactors for chromatin-modifying enzymes, linking cellular metabolism to transcriptional control. For instance, α-KG modulates the activity of histone demethylases (KDM5 and JMJD3) to orchestrate the expression profiles of maturation and proliferation genes via alterations in H3K4me3 and H3K27me3 marks. Additionally, lactate induces histone lactylation (H4K12la) to activate pro-proliferative gene networks, whereas repressor complexes containing HDAC1/2 mediate histone deacetylation to restrict the transcription of their target genes. **c** Metabolic-epigenetic coupling drives heart regeneration: The transition of cardiomyocytes (CMs) between a mature state (characterized by cell cycle arrest) and a proliferative state (characterized by cell cycle reentry) is strictly governed by these regulators. Following heart injury, shifting this balance toward favorable epigenetic regulators (Lactate, α-KG, S1P, YAP) over inhibitory factors (PDK4, Cpt1b) promotes CM proliferation and subsequent cardiac repair. Abbreviations: α-KG, α-ketoglutarate; CM, cardiomyocyte; Cpt1b, carnitine palmitoyl transferase 1B; GLUT, glucose transporter; HDAC, histone deacetylase; JMJD3, Jumonji domain-containing protein 3; KDM5, lysine demethylase 5; NMDAR, N-methyl-D-aspartate receptor; PDK4, pyruvate dehydrogenase kinase 4; S1P, sphingosine-1-phosphate; YAP, Yes-associated protein. (Figure was created with Biorender.com)

Emerging evidence indicates that distinct metabolic states determine the availability of chromatin-modifying substrates and cofactors, thereby defining whether adult cardiomyocytes remain locked in a mature oxidative identity or regain developmental plasticity. A central component of this axis is glycolytic reprogramming. Increased glucose uptake and PKM2-dependent glycolytic flux enhance biosynthetic capacity while elevating intracellular lactate [43]. Rather than serving solely as a metabolic by-product, lactate acts as a signaling metabolite that reinforces a regenerative state [98]. Lactate-mediated protein lactylation of mitochondrial enzymes such as PDHA1 and CPT2 restricts oxidative substrate entry, suppresses excessive mitochondrial respiration and shifts cells toward a synthetic metabolic configuration [204, 205]. In parallel, histone lactylation marks such as H4K12la associate with the activation of cell-cycle regulators, stabilizing a chromatin landscape permissive for proliferation. Through this mitochondrial–nuclear mechanism, glycolytic remodeling promotes cardiomyocyte dedifferentiation and cell-cycle reactivation [50, 100, 130]. In contrast, fatty acid oxidation establishes a maturation-imposed epigenetic barrier. Oxidative metabolism sustains tricarboxylic acid cycle flux and α-ketoglutarate production, which directly fuels α-KG–dependent dioxygenases including KDM5 and JMJD3 [50, 101]. These demethylases reshape H3K4me3 breadth and remove H3K27me3 repression. During postnatal maturation, this metabolic–epigenetic axis stabilizes structural gene expression programs and enforces cell-cycle exit. Experimental modulation of FAO or α-KG availability rebalances demethylase activity, attenuates maturation gene signatures and reopens proliferative chromatin domains, thereby enhancing regenerative capacity after injury [50, 101]. Lipid-derived nuclear signaling provides an additional layer of chromatin regulation. Sphingosine kinase 2 generates nuclear sphingosine-1-phosphate, which directly inhibits HDAC1 and HDAC2, leading to increased H3K9 and H3K27 acetylation [41]. This acetylation-dependent activation of *Erbb4* and *Mef2* transcriptional networks facilitates cardiomyocyte plasticity. These findings illustrate that certain lipid metabolites function as endogenous epigenetic modulators rather than merely structural components of membranes. Finally, inflammatory lipid mediators integrate immunometabolism with regenerative transcription. Macrophage-derived thromboxane A2 activates cardiomyocyte TP receptors and converges on YAP signaling. As YAP activity is sensitive to both metabolic and chromatin context, this pathway coordinates extracellular lipid cues to transcriptional programs that drive cell-cycle re-entry (Fig. 5) [42].

Collectively, these mechanisms focus on a unified principle and metabolic flux that determines chromatin enzyme activity, which in turn determines the accessibility of proliferating gene networks. Therefore, the regenerative capacity of the heart stems from the epigenetic environment permitted by metabolism rather than from the transduction of growth factors alone.

### Regulatory elements of metabolic axis for cardiac repair and regeneration

#### Non-coding RNAs

Non-coding RNAs, encompassing microRNAs (miRNAs), long non-coding RNAs (lncRNAs) and circular RNAs (circRNAs) have emerged as central coding layers of cardiomyocyte proliferation and cardiac regenerative capacity [206–209]. Collectively, these molecules define a multilayered regulatory architecture in which some species re-engage developmental cell-cycle programmers, whereas others reinforce the postnatal block to CM proliferation and consolidate terminal differentiation.

##### MicroRNAs

Developmental and injury-responsive microRNAs constitute a major regulatory axis that shapes cardiomyocyte proliferative capacity. The embryonic miR-302/302–367 cluster was first characterized in mouse myocardial infarction models, where cardiac overexpression or transient systemic delivery suppresses the Hippo kinases Mst1, Lats2 and Mob1b, activates YAP and increases the number of BrdU +, PH3 + and Aurora-B + cardiomyocytes, with associated reductions in infarct size and improved systolic function [210, 211]. Mesenchymal stem cell-derived exosomes were engineered with a cardiomyocyte-targeting peptide and loaded with miR-302. These modified exosomes are efficiently internalized by cardiomyocytes and successfully attenuate ischemia–reperfusion injury in rodent and porcine models, demonstrating the feasibility of deploying this developmental module in large-animal settings [212]. A related pro-regenerative program is mediated by the miR-17–92 cluster, as cardiac-specific loss-of-function models display reduced cardiomyocyte cell-cycle activity, whereas gain-of-function models exhibit enhanced proliferation from embryonic development through adulthood, in part through direct repression of PTEN and amplification of PI3K–AKT signaling [213].

Several individual microRNAs further illustrate distinct modes of pro-regenerative landscape. miR-199a-3p promotes cardiomyocyte cell-cycle re-entry by targeting TAOK1, β-TrCP and Cofilin-2 in neonatal rodent cardiomyocytes, which stabilizes and nuclearizes YAP while maintaining F-actin organization [214]. Delivery of miR-199a-3p using adeno-associated viral vectors in mouse and pig infarction models reduces scar burden and increases cardiomyocyte proliferation, although prolonged expression has been linked to malignant arrhythmias, highlighting the need for tight temporal control [215]. miR-590-3p enhances proliferation by repressing HOPX, HOMER1 and CLIC5, and delivery via AAV9 or exosomes to infarcted mouse hearts increases ventricular wall thickness, augments markers of cell-cycle re-entry and reduces fibrosis, with measurable improvements in ventricular performance [216, 217]. miR-21-5p integrates survival, angiogenesis and modest proliferative activity within a single regulatory axis. Telocyte-derived exosomes enriched in miR-21-5p suppress Cdip1 and downstream caspase signaling, which limits endothelial apoptosis and enhances neovascularization after infarction [218], whereas cardiomyocyte-derived exosomes carrying miR-21-5p additionally repress Spry1 and PDCD4, thereby facilitating cardiomyocyte cell-cycle activity on top of pro-survival and pro-angiogenic effects [219]. In contrast, miR-125a-5p operates primarily as a cardioprotective factor, since systemic administration in mouse and pig models reduces cardiomyocyte death and inflammatory injury without evident arrhythmogenic or off-target toxicity, preserves viable myocardium and indirectly favors regenerative remodeling even though it is not strongly mitogenic [220].

In parallel with these pro-regenerative circuits, a group of microRNAs functions as endogenous brakes on cardiac regeneration and consolidates the postnatal loss of proliferative potential [221]. Members of the miR-15 family, including miR-195, and miR-34a are the best-characterized examples, with expression rising sharply after birth in temporal concordance with cardiomyocyte cell-cycle exit and terminal maturation. The miR-15 family directly represses cell-cycle regulators such as Chek1, and inhibition of this family in neonatal mouse hearts maintains cardiomyocyte proliferation and prolongs the regenerative window after myocardial infarction [221, 222]. miR-34a targets pro-survival and pro-plasticity factors including PNUTS and SIRT1, and antagonism of miR-34a in adult infarction models attenuates apoptosis, limits adverse remodeling and improves ventricular function [223]. Taken together, these findings place developmental and injury-responsive microRNAs along a continuum that spans strong mitogenic drivers, survival- and microenvironment-modulating factors and postnatal inhibitory modules, and they identify both enhancement of pro-growth microRNAs and relief of inhibitory microRNAs as complementary entry points for therapeutic control of cardiomyocyte proliferation.

##### LncRNAs

LncRNAs operate at a higher regulatory tier by reshaping transcriptional programs and chromatin states, and by acting as competing endogenous RNAs. lncExACT1 modulates the DCHS2–Hippo–YAP axis and thereby influences the balance between physiological, exercise-like cardiac growth and pathological hypertrophy, with downstream consequences for CM growth patterns and regenerative potential. Other lncRNAs, such as Snhg1, drive self-reinforcing transcriptional networks that promote CM proliferation and enhance repair after injury, whereas lncRNAs including CAREL, CPR and CRRL have been identified as negative regulators whose silencing augments endogenous regenerative responses [224–227]. lncRNAs can also act through non-myocyte compartments. The cardiosphere-derived cell exosomal lncRNA BCYRN1 functions as a sponge for multiple miRNAs in regulatory T cells, augmenting ATG7-dependent autophagy, CCR6-mediated homing and IL-10 production; in infarction models, this BCYRN1-dependent Treg program dampens inflammation and creates a microenvironment more permissive to regenerative healing [228].

##### CircRNAs

Among circRNAs, circNfix currently provides the most compelling evidence for a circRNA that directly restrains adult myocardial regeneration. circNfix promotes proteasomal degradation of Ybx1 and suppresses miR-214 activity, thereby enforcing a high threshold for CM proliferation. In adult mouse infarction models, depletion of circNfix stabilizes Ybx1, enhances miR-214 signaling, increases CM proliferation and survival, boosts angiogenesis and leads to robust structural and functional recovery [229]. This work offers rare proof-of-principle that targeting a single circRNA can elicit a bona fide regenerative response in the adult mammalian heart.

Taken together, these non-coding RNA programs suggest that targeting pro-growth signals and relieving inhibitory circuits can be integrated with metabolic and signaling-based strategies to enhance cardiomyocyte regenerative potential. Rather than acting as solitary drivers, non-coding RNAs function as tractable nodes within a multilayered regulatory architecture, offering complementary entry points for therapeutic manipulation of cardiac regeneration.

#### Signal transduction pathways

##### PI3K/Akt/mTOR signaling pathway

The PI3K/AKT/mTOR signaling pathway is a crucial regulator of proliferation, growth, and metabolism and plays pivotal roles in cancer, diabetes, and immune responses [230]. This pathway is primarily activated by membrane receptors, including receptor tyrosine kinases (RTKs), such as EGFR and IGF-1R; G Protein-Coupled Receptors (GPCRs); cytokine receptors via JAK-STAT signaling; and integrins through FAK/Src-mediated activation. Upon activation, PI3K phosphorylates PIP2 to produce PIP3, which recruits AKT to the membrane. The full activation of AKT is then achieved via phosphorylation at the Thr308 site by PDK1 and at the Ser473 site by mTORC2. Activated AKT then regulates diverse downstream effectors, including mTORC1 (which promotes protein synthesis), GSK-3β (which modulates metabolism), FoxO transcription factors (which suppress apoptosis) and NF-κB (which enhances cell survival) [231]. The key transcription factors controlled by this pathway include FoxO family members, NF-κB, HIF-1α (which regulates angiogenesis), and MYC (which drives cell cycle progression).

The PI3K/AKT/mTOR pathway directly governs the cellular reprogramming and proliferative responses essential for regeneration [232]. As a large cell population of the heart, fibroblasts are the first responders after cardiac injury, thus making them an ideal source of cardiomyocytes. By reversing the core molecules that function in the PI3K pathway, researchers have reprogrammed nonmusical cells into cardiomyocytes [233, 234]. In combination with the core cardiac transcription factors known as Gata4, Hand2, Mef2c and Tbx5 (collectively designated as GHMT), Akt1 markedly accelerates the conversion of induced cardiomyocytes (iCMs) from fibroblasts [235, 236]. Research has demonstrated that IGF1 and PI3K act upstream of Akt, whereas the mitochondrial target of mTORC1 and forkhead box O3 (FoxO3a) act downstream of Akt to facilitate reprogramming in the generation of iCMs [237]. In parallel, sustained ErbB2 activation reactivates the cardiomyocyte cell cycle via the PI3K/Akt/mTOR pathway in developmental stages, wherein Akt orchestrates crosstalk with the ERK and GSK-3β/β-catenin pathways to enable dedifferentiation and postinjury proliferation [238]. Moreover, hypoxia-induced integrin β3 suppresses apoptosis and stimulates proliferation through the modulation of PTEN/Akt/mTOR and ERK1/2, which frequently cooperate with integrins αv/β1 [239].

The PI3K/Akt/mTOR signaling pathway also closely integrates metabolites with growth signals to align with cardiomyocyte proliferation by controlling energy status and endocrine homeostasis. The metabolic-growth interplay is robustly exemplified by key cardiac repair regulators and hormones. A previous study demonstrated that thyroid hormone (TH) stimulates cellular proliferation, differentiation, and maturation through the IGF-1-dependent PI3K/AKT/mTOR signaling pathway [240]. In neonatal mouse models, thyroxine levels remain low but significantly increase during myocardial development [241, 242]. This surge in thyroid hormone levels temporally coincides with the burst of cardiomyocyte proliferation that occurs around preadolescence (Postnatal day 15) [241]. However, contrasting evidence indicates that postnatal cardiomyocytes primarily respond to TH via cellular hypertrophy rather than through proliferation [243]. Although these findings do not elucidate the precise mechanisms of TH-mediated cardiomyocyte proliferation, future studies should prioritize the tracking of cell proliferation in vivo and emphasize the need for more sophisticated approaches.

In addition to hormones secreted by endocrine glands, hepatic factors actively regulate the Akt pathway in distal cells, thereby constituting a key regulatory mechanism in regeneration. In the liver, insulin and insulin-like growth factor (IGF) activate the PI3K/Akt pathway to inhibit apoptosis via Akt-mediated mTORC1 activation, thereby resulting in S6K1 and 4E-BP1 phosphorylation, which drives protein synthesis [244]. Similar to TH, early studies have demonstrated a rapid increase in systemic Igf1 concentrations after birth [245]. Clinical studies have suggested that TH directly modulates Igf1 levels; more recently, in vivo and in vitro studies have confirmed that T3 increases the expression of Igf1 and Igf1R in cardiomyocytes, thus eventually resulting in increased phosphorylation of the PI3K and AKT proteins [244]. In addition to its regulatory role in the PI3K pathway, insulin facilitates GLUT4 translocation to augment glucose uptake, which indirectly contributes to glucose-dependent activation of the PI3K pathway [246]. Moreover, enhanced fatty acid metabolism flux in cardiomyocytes reduces cardiac regeneration, whereas the adipokine leptin significantly regulates the PI3K/Akt pathway via lipid metabolic modulation [247, 248]. Research has demonstrated that LepRb/JAK2 signaling modulates fatty acid utilization and induces cardiomyocyte hypertrophy in vitro [249, 250].

Utilizing a distinct mechanism compared to hormones, bioactive small molecules also regulate cardiac proliferation through the PI3K/Akt/mTOR signaling pathway. A previous study demonstrated that the branched-chain amino acid leucine can entirely bypass the PI3K/Akt axis, thereby directly activating mTOR via its mitochondrial axis to provide essential metabolic regulation and consequently inducing pathological cardiac hypertrophy associated with chronic PI3K/Akt hyperactivation [251]. Additionally, intermediates catalyzed by lipid metabolism, such as the sphingolipid metabolite known as dihydrosphingosine-1-phosphate (DHS-1-P), leverage PI3K/Akt/mTOR signaling to influence collagen synthesis and hypertrophy, thus implicating this metabolite in fibrotic repair dynamics [252].

Collectively, these mechanisms involve various metabolic regulators (such as TH, insulin/IGF-1, leptin, leucine and DHS-1-P) that act to direct cell fate modulators (involving Akt1 reprogramming, ErbB2 proliferation, and integrin β3 survival), which collectively demonstrate the PI3K/Akt/mTOR pathway as a central regulatory hub that synchronizes cardiac repair with metabolic capacity. Emerging factors such as the purine synthesis enzyme Adssl1 (which activates mTORC1 to promote cardiomyocyte proliferation) further support this metabolic-regenerative paradigm, thereby underscoring the importance of the PI3K/Akt/mTOR pathway and its inherent therapeutic significance in heart regeneration.

##### Hippo/YAP signaling pathway

The Hippo signaling pathway is a pivotal growth control mechanism observed in multicellular organisms that can coordinate cell proliferation and differentiation, thereby regulating organ development and tissue regeneration [253]. The biological function of the Hippo pathway is highly conserved in mammals and *Drosophila*. In mouse models, the genetic deletion of Hippo pathway genes leads to significant enlargement of affected organs [254, 255].

Specifically, the Hippo-YAP signaling pathway is a linear kinase cascade that modulates cell proliferation and apoptosis by inhibiting the core transcription coactivator known as yes-associated protein (YAP) and its homolog known as WW domain-containing transcription regulator 1 (WWTR1 or TAZ). Upon the activation of the Hippo pathway, YAP undergoes phosphorylation and subsequently stagnates in the cytoplasm or is degraded, thereby suppressing its transcriptional activity [256]. Conversely, the silencing of the Hippo pathway or the nuclear translocation of YAP can promote cell proliferation, and this phenomenon is particularly evident in embryonic and neonatal hearts [257].

The activation of YAP, which is the core effector molecule of the Hippo pathway, promotes cardiomyocyte proliferation and inhibits apoptosis. The adult mouse heart exhibits a limited capacity for generating new cardiomyocytes following injury. Notably, adult mouse cardiomyocytes that are deficient in key downstream effectors of the Hippo signaling pathway, such as Salvador homolog 1 (SAV1) or large tumor suppressor kinases (LATS1/2), exhibit a significantly increased capacity to re-enter the cell cycle [258, 259]. Moreover, Sav1-deficient hearts exhibit enhanced cardiac regeneration with reduced fibrosis in cardiac apex resection models at postnatal day 8 (P8) [260]. These findings indicate that the Hippo signaling pathway plays a crucial role in regulating cardiomyocyte proliferation and cardiac regeneration.

Subsequent studies have revealed that YAP expression re-emerges in the border zone of myocardial infarction, which is potentially associated with changes in extracellular matrix stiffness [261]. Similarly, Xin et al. demonstrated that YAP transgenic mice (the αMHC-Cre model) exhibit enhanced cardiac regeneration following left anterior descending (LAD) coronary artery occlusion, with reduced fibrosis and improved heart functional parameters (such as cardiac output and ejection fraction) being observed [262]. Consistent with this finding, the widely prescribed β1-adrenergic receptor (β1-AR) blocker metoprolol enhances cardiac repair after myocardial infarction via the stimulation of the α-stimulating (GNAS)-Ras homolog family member A (RhoA)-YAP signaling pathway [263].

The cardiac regenerative potential of YAP/TAZ is mediated through diverse molecular mechanisms. In addition to stimulating cell cycle progression and survival-related gene expression, YAP activates IGF1-Akt signaling and upregulates fetal cardiac genes, including actinin alpha 1 (*Actn1*) [262]. Additionally, YAP modulates cytoskeletal reorganization by regulating key molecules such as actin-related protein T2 (ACTRT2), plakophilin 4 (PKP4), enabled homolog (ENAH), and formin 2 (FMN2) [264]. Importantly, the genetic ablation of YAP/TAZ leads to impaired cardiac immune homeostasis, which is characterized by diminished regulatory T-cell infiltration and IFN-γ downregulation in the infarcted myocardium. These immunological disturbances exacerbate post-MI pericardial inflammation and fibrotic remodeling, thereby ultimately leading to lethal cardiomyopathy [265].

The metabolic status is closely associated with cellular growth and proliferation, whereas the Hippo signaling pathway is highly sensitive to the cellular energy state. For example, the inhibition of glucose metabolism (2-DG) induces YAP/TAZ phosphorylation via AMP-activated protein kinase (AMPK)-mediated LATS1/2 activation. AMPK directly phosphorylates YAP at Ser94, thus disrupting its interaction with TEAD1–4 [266, 267]. Conversely, under high-glucose conditions, O-GlcNAcylation (which is a glucose-dependent modification fueled by uridine diphosphate glucose [UDP-G] generated from the hexosamine biosynthesis pathway [HBP]) stabilizes YAP by inhibiting its degradation (particularly through Thr241 modifications) to promote cell growth [268]. High-glucose conditions further amplify this axis by enabling O-GlcNAcylation of Hippo components such as angiomotin (AMOT) and homeodomain-interacting protein kinase (Hipk), thus reinforcing proliferative signaling [269, 270]. Although validated in oncology and murine embryogenesis, the role of O-GlcNAcylation in cardiac regeneration remains unexplored [271]. Within the glycolytic context, PFK-1 forms nuclear complexes with TEAD1 and YAP to potentiate transcriptional activation ability.

In addition to the cholesterol-lowering effects mediated by the inhibition of 3-hydroxy-3-methylglutaryl coenzyme A (HMG-CoA) reductase, statins have emerged as promising therapeutic targets in regenerative medicine research due to their ability to activate the Hippo pathway. By inhibiting the mevalonate pathway to block YAP activation, statins deplete geranylgeranyl pyrophosphate (GGPP), which is essential for Rho GTPase-driven cytoskeletal remodeling [272, 273]. In addition to pharmaceutical intervention, key enzymes and metabolites of lipid metabolism play critical regulatory roles in the Hippo-YAP signaling pathway. Moreover, stearoyl-CoA desaturase 1 (SCD1) accelerates proliferation via lipogenesis, including the generation of phospholipids, triglycerides, and cholesteryl esters [274]. The knockdown or pharmacological inhibition of SCD1 impairs YAP nuclear localization and transcriptional output [275, 276]. Similarly, the depletion of SCD1 enhances the conversion of fibroblasts to iCMs and iCM reprogramming [203]. Palmitic acid exhibits dual regulation, including the induction of MST1 expression via mtDNA/cGAS-STING/IRF3 signaling to inactivate YAP while simultaneously enhancing YAP-TEAD binding through NF2-controlled palmitoylation of TEAD transcription factors [277]. Palmitoylation modulated by cell density via FAS and acetyl-CoA carboxylase expression stabilizes TEADs to form the YAP-TEAD complex [278, 279].It is evident that following heart damage, the metabolic regulation of the YAP/TAZ pathway not only induces cardiomyocyte proliferation but also modulates immune cell responses to facilitate heart regeneration. Research has demonstrated that macrophage-derived metabolites such as thromboxane A2 and OSM additionally contribute to postinjury YAP activation [280].

Beyond its established role in regulating cell proliferation and regeneration, YAP signaling has also emerged as an important modulator of ferroptosis, a form of iron-dependent regulated cell death that contribute to cardiomyocyte loss after myocardial infarction. In neonatal and juvenile mouse MI models, ischemic cardiomyocytes were reported to undergo ferroptosis rather than classical apoptosis or necroptosis. Cardiomyocyte-derived Pitx2, a YAP-interacting transcriptional cofactor, was identified as an important regulator of this process, suppressing ferroptosis and limiting adverse fibrotic remodeling through modulation of ferroptosis-associated gene expression [281]. In cancer models, YAP activation has been shown to promote ferroptosis susceptibility, in part through transcriptional regulation of ferroptosis-associated genes such as* Acsl4* and *Tfrc* [282]. In contrast, in the injured myocardium, Pitx2 cooperatively regulates antioxidant and mitochondrial oxidative phosphorylation programs with YAP and has been shown to inhibit ferroptosis in cardiomyocytes [283]. These findings suggest that effective cardiac regeneration requires not only induction of cardiomyocyte cell-cycle activity, but also preservation of cardiomyocyte survival through coordinated control of redox homeostasis and ferroptosis.

YAP activation represents one of the most potent strategies for overcoming cardiomyocyte cell-cycle arrest, but its therapeutic application requires precise control of the activation window and intervention duration. Constitutively active YAP variants such as YAP5SA, can drive adult cardiomyocytes to re-enter the cell cycle and progress through early proliferative phases, thereby supporting the regenerative potential of Hippo pathway inhibition [284]. However, excessive or sustained YAP activation may uncouple cell-cycle induction from coordinated tissue repair. In cardiomyocyte-specific YAP5SA models, robust cell-cycle reactivation has been associated with pathological cardiac growth, obstructive cardiomyopathy and reduced survival, indicating that uncontrolled proliferative stimulation can impose a substantial physiological cost [285]. These findings revealed that YAP-based regenerative strategies should be designed as temporally restricted and quantitatively titrated interventions, ideally aligned with the early post-injury repair phase, to maximize cardiomyocyte renewal while minimizing the risks of pathological hypertrophy, mitotic failure and lethal cardiac dysfunction.

The Hippo-YAP pathway does not function in isolation; rather, its crosstalk with other signaling pathways, particularly the PI3K/Akt/mTOR pathway, plays a critical role in cardiac regeneration. Mechanistically, YAP directly activates Pik3cb expression through its interaction with transcriptional enhanced associate domain (TEAD) transcription factors, thereby initiating the PI3K/Akt signaling cascade [286]. Notably, the AAV-mediated overexpression of Pik3cb can partially rescue cardiac dysfunction induced by YAP deficiency, thereby enhancing cardiomyocyte proliferation while reducing apoptosis [287]. These findings establish Pik3cb as a molecular bridge connecting the Hippo-YAP and PI3K-Akt pathways, thus collectively promoting cardiomyocyte proliferation and survival.

##### Wnt/β-catenin signaling pathway

The Wnt/β-catenin signaling pathway primarily regulates cell proliferation and differentiation by stabilizing β-catenin and facilitating its nuclear translocation, thereby activating the expression of downstream target genes such as *Ccnd1* and *Axin2*. Upon the binding of Wnt ligands to receptors such as Frizzled and LRP5/6, the β-catenin destruction complex (containing Axin, CK1α and GSK-3β) is inhibited, thus leading to β-catenin accumulation and subsequent nuclear translocation to activate transcription [288]. Both canonical Wnt signaling (β-catenin-dependent) and noncanonical Wnt signaling (β-catenin-independent) play critical roles during human cardiomyocyte development. During the differentiation of human embryonic stem cells (hESCs) into mesoderm, canonical Wnt signaling is activated through WNT3 and WNT8A via the FZD7 receptor, which regulates Branchyury (BRY) expression and promotes mesoderm formation [289]. In contrast, noncanonical Wnt signaling (particularly the JNK-dependent pathway) is activated via WNT5A and WNT5B, thus modulating MESP1 expression to facilitate cardiovascular progenitor cell specification and enhance cardiomyocyte differentiation [290].

Studies have demonstrated that the Wnt/β-catenin pathway plays a central role in cardiac regeneration models such as zebrafish and neonatal mammals. Research on zebrafish hearts has revealed increased Wnt/β-catenin signaling activity at the injury border zone following cardiac injury, with significantly increased *axin2* and *lef1* transcription observed at 7 days postinjury. Subsequent experiments revealed that either Axin1 overexpression or pharmacological inhibition (IWR-1) significantly reduces the population of proliferating cardiomyocytes [291]. Using both neonatal mice and human embryonic stem cell (ESC)-derived cardiomyocytes, researchers successfully activated the Wnt/β-catenin signaling pathway and promoted cardiomyocyte proliferation via treatment with N-cadherin antibodies and the GSK inhibitor CHIR99021. Mechanistically, the N-cadherin antibody increases cytoplasmic β-catenin levels by releasing sequestered β-catenin, thereby activating Wnt signaling. Similarly, CHIR99021 prevents β-catenin degradation through GSK inhibition, thereby increasing cytoplasmic β-catenin levels [292]. After myocardial injury, the Wnt/β-catenin signaling pathway plays a multifaceted regulatory role in cardiomyocyte survival [293]. With respect to its antiapoptotic effects, Wnt pathway activation leads to cytoplasmic β-catenin accumulation and subsequent nuclear translocation, where it binds to TCF/LEF transcription factors to upregulate antiapoptotic genes (such as *Bcl-2* and *Survivin*), thereby inhibiting cardiomyocyte apoptosis [294, 295]. In terms of promoting cell survival, the Wnt/β-catenin pathway enhances the expression of growth factors such as IGF-1 and VEGF. These factors activate the PI3K/Akt and MAPK/ERK signaling cascades to support cardiomyocyte survival [296].

Under high-glucose conditions, cardiomyocytes exhibit increased sensitivity to Wnt/β-catenin signaling, as evidenced by the upregulated expression of Wnt pathway-related genes (including *Axin2* and *Scn5a)*. This observation suggests that glucose may indirectly promote cardiomyocyte proliferation via Wnt pathway activation [297]. Furthermore, the key transcription factor known as KLF1 and the key enzyme known as PKM2 can synergistically regulate glycolysis and fatty acid metabolism, thereby modulating both mitochondrial function and Wnt/β-catenin signaling [298]. This integrated metabolic regulation process shapes a pro-regenerative condition and upregulates the expression of cell cycle-related genes (including *Ccna2* and *Ccnb1*), thus ultimately promoting cardiomyocyte proliferation and survival [299, 300].

Wnt signaling exhibits stage-dependent and cell-specific effects following myocardial infarction. The inconsistent findings regarding its beneficial or detrimental roles likely reflect differences in the temporal and spatial patterns of pathway activation during post-infarction healing. In a study using a TCF/Lef promoter-hFTH MRI reporter system, Wnt/β-catenin/TCF signaling was shown to be persistently activated in the border zone after myocardial infarction in rats [301]. Pharmacological inhibition of β-catenin/TCF-dependent transcription attenuated the Wnt reporter signal and was associated with reduced left ventricular dilatation, less deterioration of ejection fraction, and improved ventricular remodeling. Histological analysis further showed reduced infarct scar size and increased peri-infarct arteriolar density after pathway inhibition [301]. These findings suggest that sustained Wnt/β-catenin/TCF activation during specific phases of post-infarction repair may contribute to scar expansion and adverse ventricular remodeling, whereas timely inhibition of this pathway may help mitigate ischemia-induced structural remodeling.

Functional crosstalk between Wnt/β-catenin and Hippo/YAP signaling has emerged as an important regulatory axis in cardiomyocyte proliferation. Genetic studies have shown that Hippo signaling restrains cardiac growth partly by suppressing a subset of Wnt/β-catenin target genes, whereas reduced β-catenin dosage can mitigate the myocardial overgrowth caused by Hippo inactivation [302]. Conversely, YAP activation can promote β-catenin stabilization through the IGF–AKT–GSK3β axis, and YAP has been reported to interact with β-catenin to regulate growth-associated genes [303]. These findings indicate that Wnt and Hippo signaling do not operate as isolated mitogenic pathways, but instead converge on shared transcriptional and metabolic programs that shape cardiomyocyte cell-cycle activity during cardiac development and repair.

##### Notch signaling pathway

The Notch signaling pathway is essential for multicellular organismal development and tissue homeostasis; moreover, it plays a pivotal role in cardiac development and repair. Emerging evidence demonstrates its multifaceted functions in cardiac regeneration, particularly following MI. These protective and regenerative mechanisms include the protection of cardiomyocytes, promotion of angiogenesis, and facilitation of myocardial tissue regeneration. During cardiac development, Notch1 activation is observed in proliferating embryonic and immature cardiomyocytes, with subsequent downregulation being noted in the postnatal myocardium [304–306]. Intriguingly, the transient reactivation of Notch signaling occurs in adult cardiomyocytes following injury, thus suggesting its relationship with potential cardiac repair processes. Experimental studies have confirmed that the activation of the Notch1 intracellular domain (NICD) mitigates myocardial injury severity and improves cardiac hemodynamic function [307]. Conversely, the genetic ablation of Notch1 (either systemically or specifically in bone marrow-derived cells [BMDCs]) compromises postinfarction cardiac repair [308].

**Fig. 6:**
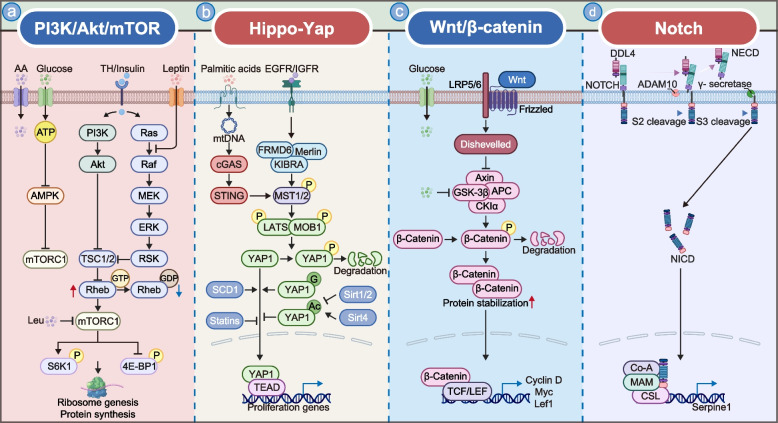
Pro-regenerative pathways in cardiomyocytes and their metabolic regulators. **a** PI3K/Akt/mTOR signaling: Extracellular nutrients and hormones (AA, Glucose, TH/insulin and leptin) activate PI3K/Akt and MAPK cascades, converging to inhibit the TSC1/2 complex. Thus activate the mTORC1 and promote ribosome biogenesis and protein synthesis via S6K1 and 4E-BP1. **b** Hippo-YAP signaling: Upstream stimuli modulate the MST-LATS core kinase cascade. Kinase activation induces YAP1 phosphorylation and subsequent proteasomal degradation. Conversely, unphosphorylated YAP1 translocate to the nucleus, interacting with TEAD to drive the transcription of proliferation-related genes. **c** Wnt/β-catenin signaling: Wnt ligand binding to receptors inhibits the GSK-3β-mediated destruction complex. This prevents β-catenin phosphorylation and degradation, allowing stabilized β-catenin to accumulate in the nucleus and associate with TCF/LEF to upregulate target genes such as Cyclin D and Myc. **d** Notch signaling: Ligand (e.g., DLL4) engagement triggers sequential proteolytic cleavages of the Notch receptor by ADAM10 and γ-secretase. The liberated Notch intracellular domain (NICD) translocates to the nucleus, assembling a transcriptional complex with CSL and co-activators to activate downstream gene expression. (Figure was created with Biorender.com)

Related studies have revealed the rapid activation of the Notch signaling pathway following cardiac injury in zebrafish. Significant upregulation of the expression of both Notch receptors (such as Notch1a, Notch1b, Notch2, and Notch3) and ligands (Dll4) occurs in the injured myocardium. During embryonic development, Notch signaling is robustly activated in the atrial endocardium. In adult zebrafish hearts, this pathway is highly expressed in both endocardial and epicardial cells. Moreover, the activation of Notch signaling in endocardial or epicardial cells demonstrates its regulation of cardiomyocyte proliferation via noncell-autonomous mechanisms [309]. Both embryonic and adult zebrafish models have demonstrated that Notch inhibition disrupts cardiomyocyte proliferation. Specifically, in adult zebrafish hearts, Notch signaling facilitates CMDD and proliferation processes that are essential for cardiac regeneration [310]. Mechanistically, this pathway enhances myocardial regeneration by downregulating plasminogen activator inhibitor-1 (*Serpine1*) expression. Although Serpine1 is initially upregulated postinjury, its expression markedly decreases during the peak cardiomyocyte proliferation phase (7 days postinjury) [311]. Interestingly, glucose may modulate Notch signaling via the O-glucosylation of Notch receptors. Studies have suggested that O-glucose modifications facilitate juxtamembrane cleavage of Notch receptors, which is a critical step for signal activation (Fig. 6) [312].

## Emerging therapeutic approaches for cardiac repair and regeneration

**Fig. 7:**
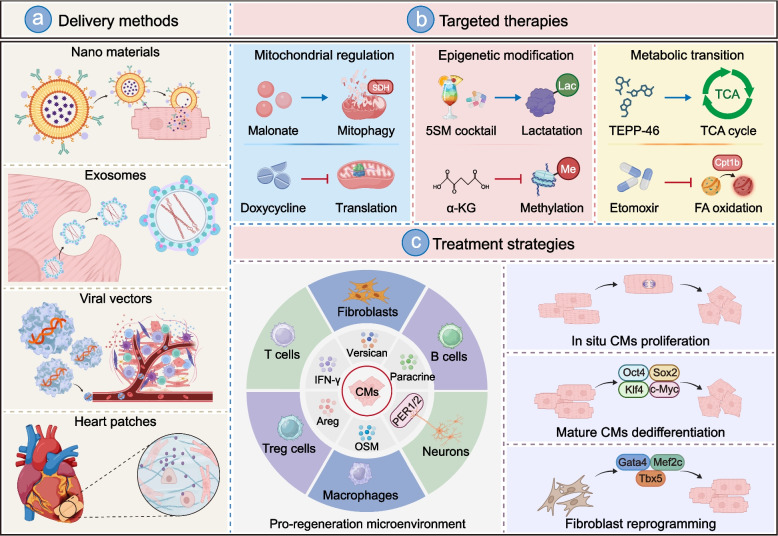
Therapeutic strategies and delivery platforms for heart regeneration. **a** Delivery method: Various delivery vehicles, including nanomaterials, exosomes, viral vectors, and engineered heart patches, are utilized to effectively transport therapeutic agents to the myocardium. **b** Targeted therapies: Pharmacological interventions modulate specific cellular networks. Mitochondrial regulation involves malonate-induced mitophagy and doxycycline-mediated inhibition of mitochondrial translation. Epigenetic modifications are manipulated via 5SM cocktail-induced lactylation and α-KG-dependent inhibition of methylation. Metabolic transitions are driven by TEPP-46 and etomoxir, which suppresses fatty acid (FA) oxidation by inhibiting Cpt1b. **c** Treatment strategies: Regenerative approaches involve microenvironmental modulation and cellular reprogramming. The pro-regenerative microenvironment is orchestrated by paracrine crosstalk between cardiomyocytes (CMs) and non-myocytes (e.g., fibroblasts, macrophages, T/B cells, neurons) via factors such as Versican, IFN-γ, Areg, and OSM. Cellular strategies aim to replenish CMs through direct induction of CM proliferation, dedifferentiation of mature CMs utilizing Yamanaka factors (Oct4, Sox2, Klf4, c-Myc), or direct reprogramming of cardiac fibroblasts into CM-like cells using core transcription factors (Gata4, Mef2c, Tbx5). Abbreviations: α-KG, α-ketoglutarate; Areg, amphiregulin; CM, cardiomyocyte; Cpt1b, carnitine palmitoyl transferase 1B; FA, fatty acid; IFN-γ, interferon gamma; OSM, oncostatin M; PER1/2, period circadian regulator 1/2; TCA, tricarboxylic acid. (Figure was created with Biorender.com)

Cardiac regeneration research is rapidly shaping into a multidimensional therapeutic framework. As our understanding of cardiomyocyte metabolism, cellular plasticity, and the remodeling of the microenvironment caused by injury continues to grow, various interventions are being integrated into a coherent system. These strategies cover the aspects of intracellular regulation, intercellular signaling and tissue engineering, which together constitute the translational application category of modern cardiac regenerative medicine. This section provides a systematic overview of these main modules and evaluates their effectiveness for preclinical and translational applications, delineating the current architecture of regenerative therapies (Fig. 7).

In this context, metabolic reprogramming maintains proliferative capacity and enhances cardiac function by regulating glycolysis, lipid utilization, and mitochondrial redox balance. At the same time, transcriptional reprogramming and iPSCs based therapies aim to induce cell lineage switching or to supplement missing cardiomyocytes, thereby promoting functional recovery in both acute and chronic models. For paracrine regulation, extracellular vesicles provide powerful platforms to stimulate angiogenesis, reduce apoptosis, and control inflammation. At the structural level, injectable hydrogels and heart patches optimize local microenvironments to enhance cell retention and tissue integration, while nanoengineered biomaterials provide precise spatiotemporal control of load delivery. By presenting these approaches in parallel, we construct a comprehensive therapeutic map that enables cross-strategy comparisons and lays the foundation for future combined approaches (Table 3).

**Table 3:** Pre-clinical strategies and clinical readiness for cardiac regeneration

Vehicle	Components	Delivery methods	Targets	Outcomes	TRL grade (1–9)	Reference
AAV	Sav-shRNA	Catheter-mediated subendocardial injection (NOGA-guided intramyocardial injection)	Inhibit Hippo kinase cascade to suppress YAP/TAZ-TEAD transcriptional program	Induces CM cell-cycle activity/division; reduces scar; increases capillary density; improves LVEF	6	[249]
	circNfix	Direct intramyocardial injection (mouse MI models)	Inhibit anti-proliferative programs (circNfix-Ybx1/ubiquitin–proteasome pathway)	Increases CM proliferation markers and improves repair phenotypes in preclinical models	4	[219]
	circHipk3	Direct intramyocardial injection (mouse MI models)	Notch1 signaling and proliferative programs	Enhances CM proliferation readouts; improves functional recovery in MI models	4	[298]
	circCDYL	Direct intramyocardial injection (mouse MI models)	circRNA-miRNA-mRNA axis controlling CM proliferation	Promotes CM cell-cycle activity and improves cardiac repair phenotype	4	[299]
Citrate-saline biologically compatible buffer	VEGF-A mRNA	Intracardiac injection (porcine MI models)	Angiogenesis and pro-repair vascular remodeling	Improves cardiac function in sub-acute window; supports translation of mRNA therapy	5	[300]
Sucrose citrate buffer	PKM2-modRNA	Intramyocardial injection (mice and porcine models)	Pkm2 directly interacts with β-catenin, upregulates its downstream targets (Cyclin D1 and C-Myc)	Increases expression of cell-cycle promoting genes and cell cycle markers in postnatal CMs	5	[43, 95]
Small extracellular vesicles (sEVs)	Bioactive cargo (contains miR-486-5p)	Systemic administration (mouse MI models)	Fibroblast MMP19–VEGFA axis; pro-angiogenic signaling	Promotes angiogenesis; improves cardiac recovery without increased arrhythmia in NHP model	4	[301]
CDC-CMs	Cardiospheroids	Intramyocardial injection (mouse MI models)	Cell replacement and paracrine signaling supporting repair	Improves cardiac function and remodeling outcomes in preclinical models	5	[302]
CDC-CMs derived EVs	Bioactive cargo (contains miR-146a)	Intramyocardial injection (mouse MI models); intracoronary injection (porcine MI models)	miR-146a to regulate systolic pathway	Inhibit apoptosis and promote proliferation of cardiomyocytes, while enhancing angiogenesis	5	[303, 304]
	Bioactive cargo (contains miR-181b)	Intramyocardial injection (mouse MI models)	miR-181b to suppress inflammatory macrophage responses	Polarize macrophages from pro-inflammatory to anti-inflammatory state	4	[305]
	Bioactive cargo	Pericardial administrations (mouse MI models)	Immune regulation to stimulate M2 polarization	Polarize macrophages from pro-inflammatory to anti-inflammatory state	4	[306]
hiPSC derived CM-EVs	Functionally validated cargo	Intramyocardial injection at the peri‐infarct region (mouse MI models)	Rapamycin (mTOR) signaling pathway and autophagy to repair (paracrine signaling)	improve the systolic and LVEF function and enhance myocardium viability	4	[307]
	Bioactive cargo	Intramyocardial injection (rat MI models)	Angiogenesis (miR218-5p, miR122-5p, miR-25–3 e.g.,); antifibrosis (miR-1, miR-24, miR-25-3p, and miR-218-5p); M2 macrophage polarization (miR-21, miR-92, and miR-320a)	Suppress cell death and enhance cardiac function; angiogenesis; reduce the fibrosis; promote M2 polarization	4	[308]
	Bioactive cargo	Intramyocardial injection (swine MI models)	Cellular creatine kinase metabolism (paracrine signaling)	Promote angiogenesis, proliferation, cell survival, and the expression of pro migratory molecules	5	[309]
Collagen patches encapsulated with the hiPSC derived CM-EVs	Bioactive cargo	Patch transplantation after LAD ligation (rat MI models)	Paracrine signaling	Reduce apoptosis and pathological hypertrophy	5	[310]
CPC-EVs	Bioactive cargo (contains miRNA cluster miR-144/451)	Intramyocardial injection (mouse I/R models)	miR-451 to regulate apoptosis	Reduce apoptosis and the production of inflammatory immunoglobulin	4	[311, 312]
human CPC-EVs	Bioactive cargo	Intracoronary administration (NOGA-guided) in porcine MI models	Paracrine signaling	Improve the LVEF and reduce fibrosis	5	[313]
Injectable hyaluronic acid hydrogel	miR-302	Intramyocardial injection (mice MI models)	miR-302 to inhibit the Hippo-YAP pathway	Increase CM proliferation markers and improve cardiac functions	4	[199]
Microspheres (microgels and AR-Neo-EVs)	Bioactive cargo (contain overexpressed Wdr75)	Intramyocardial injection (mice MI models)	Overexpress Wdr75 to regulate *P53*	Increase CM proliferation markers and improve cardiac functions	4	[314]
7AP-collagen hydrogel	HDAC-7	Intramyocardial injection (mice MI models)	Reduce apoptosis and stimulate CM cell cycle progression	Increase CM proliferation markers and improve cardiac functions	4	[315]
rhCHK1-hydrogel	rhCHK1	Intramyocardial injection (porcine MI models)	Reduce apoptosis and fibrosis; stimulate CM cell cycle progression	Increase CM proliferation markers and improve cardiac functions	5	[316]
CgNPs	BIO/IGF-1	Intramyocardial injection (rat MI models)	Stimulate CM cell cycle progression	Increase CM proliferation markers and improve cardiac functions	5	[317]
Multifunctional hydrogels (CMC)	Curcumin (Cur) and tailored recombinant humanized collagen type III (rhCol III)	Intramyocardial injection (rat MI models)	Reduce apoptosis, improve anti-inflammatory response; pro-angiogenesis effect and stimulate CM cell cycle progression	Increase CM proliferation markers and improve cardiac functions	5	[318]
Polyphenol reinforce peptide glycocelyx-like coating on cardiac occluders	Carboxylated chitosan, epigallocatechin-3-gallate (EGCG), tanshinone IIA sulfonic sodium (TSS), and hyaluronic acid	Intramyocardial injection (New Zealand white rabbit carotid artery models)	Reduce thrombosis by promoting endothelialization, reduce apoptosis, improve anti-inflammatory response and stimulate CM cell cycle progression	Increase CM proliferation markers and improve cardiac functions	5	[319]
The pMXs-DsRed Express retrovirus	GMT	Intramyocardial injection (mice MI models)	Activate developmental transcription factors	Induce cardiomyocyte-like cells from postnatal cardiac fibroblasts	3	[223]
Retroviral vector pBabe-X	GHMT	Intramyocardial injection (mice MI models)	Activate developmental transcription factors	Induce cardiomyocyte-like cells from postnatal cardiac fibroblasts	3	[320]
Doxycycline induced expression system	OSKM	Genetic engineering	Activate pluripotency transcription factors	Induce cardiomyocyte-like cells from postnatal cardiac fibroblasts	3	[146]
Fibroblast growth medium with CRFVPT	CRFVPT (CHIR99021, RepSox, Forskolin, VPA, Parnate, and TTNPB)	Cell culture	Activate cardiac developmental program	Induce cardiomyocyte-like cells from postnatal cardiac fibroblasts	3	[321]
Lentiviral vectors	9 C (CHIR99021, A83-01, BIX01294, AS8351, SC1, Y27632, OAC2, SU16F, and JNJ10198409)	Intramyocardial injection (mice MI models)	Activate cardiac developmental program	Induce cardiomyocyte-like cells from postnatal cardiac fibroblasts	3	[322]
Collagen patches	Isolated CMs from neonatal rats	Patch transplantation after LAD ligation (rat MI models)	Exogenous cardiomyocyte supplementation	Improve cardiac functions	5	[323]
Collagen patches	ESC-CMs	Patch transplantation (rat I/R models)	Exogenous cardiomyocyte supplementation	Improve cardiac functions	5	[324]
Fibrin patches	hiPSC-CMs	Patch transplantation (guinea pig cryoinjury models)	Exogenous cardiomyocyte supplementation	Improve cardiac functions	5	[325–327]
Engineered heart tissue	hiPSC-CMs	Patch transplantation (rat I/R models and healthy rats)	Exogenous cardiomyocyte supplementation	Improve cardiac functions	5	[328–330]
Engineered heart tissue	hiPSC-CMs	Patch transplantation (pig I/R models)	Exogenous cardiomyocyte supplementation	Improve cardiac functions	5	[61]
Engineered heart tissue	hiPSC-CMs	Patch transplantation (rhesus macaques chronic heart failure models and bridge-to-transplant human)	Exogenous cardiomyocyte supplementation	EHT remuscularization and improve cardiac functions	6	[331]

*TRL* Technology Readiness Level

### Metabolic therapies

Metabolism represents a pivotal regulatory axis in cardiac regeneration [130]. Genetic manipulation and other studies have established the core features of the pro-regenerative metabolic phenotype: vigorous anabolism, adequate energy supply, and low levels of mitochondrial stress [6]. However, reshaping this pro-regenerative metabolic flux faces significant challenges in terms of systematisms and multi-factor synergy. Currently, preclinical attempts are mostly limited to interventions for single metabolites or local pathways (Table 2) [313–315]. To bridge this gap, future therapies must account for systemic circulation and holistic metabolic remodeling. On the basis of this consensus, we systematically identified the clinically approved and proven cardiovascular metabolic drugs. By integrating their systemic metabolic regulatory effects with the latest research on regeneration targets, we aimed to construct a candidate repertoire of drugs for cardiac regeneration with high clinical translational potential.

#### Targeting glucose metabolism in cardiac repair and regeneration

Pharmacological enhancement of glycolysis reflects a fundamental strategy for reinstating developmental metabolic features. By encoding a key glycolytic enzyme, PKM2 serve as metabolic intervention that reactivates the cardiomyocyte cell cycle via enhanced glycolysis, effectively reversing heart failure and restoring cardiac function [43]. Similarly, activation of the thromboxane A₂ receptor with U-46619 drives glycolytic metabolism and proliferative modulation, whereas antagonists such as S18886 and SQ29548 blunt these effects [42].

Translation of this metabolic principle into the myocardium has begun to yield regenerative signals. A five-compound metabolic cocktail (5SM) induces adult cardiomyocyte renewal by activating the lactate–LacRS2–mTOR axis and reprogramming fuel preference toward glycolysis, improving ventricular function after myocardial infarction [205]. In parallel, succinate dehydrogenase (SDH) inhibition with malonate esters or Atpenin A5 reduces injury-associated ROS levels, induces a glycolytic shift and promotes cardiac regeneration in adult mice [100]. Supplementation with metabolic intermediates such as α-ketoglutarate or lactate reduces infarct scar burden and preserves contractile performance [101, 121].

Manipulation of the PDK–PDH axis further influences glycolytic commitment. Enhanced PDK activity increases lactate-linked flux, precedes YAP activation and supports cardiomyocyte dedifferentiation in zebrafish and rodent regenerative models [51, 316]. Delivery of nucleus-targeted GLP-1 via ultrasound-targeted microbubble destruction suppresses FoxO1 activity, induces cyclin D1 activity and increases proliferation in rat post-MI myocardium [317]. These studies position PDK activation, glycolytic reinforcement and metabolite supplementation as convergent levers for reinstating a neonatal-like metabolic state permissive for cardiomyocyte cell-cycle re-entry [317].

#### Targeting lipid metabolism and ketone use in cardiac repair and regeneration

Suppressing excessive fatty-acid oxidation (FAO) reduces metabolic rigidity and enhances glucose utilization. The CPT1 inhibitor etomoxir improved ventricular remodeling in rodent MI models [50] and improved haemodynamics in an ERGO heart-failure trial before hepatotoxicity stopped further development [318]. Trimetazidine, a partial inhibitor of mitochondrial long-chain 3-ketoacyl-CoA thiolase (LC-3KAT), reduces fatty-acid oxidation and thus stimulates glucose oxidation, improving functional recovery/ischemia tolerance in experimental models [319–321].

Beyond bulk FAO flux, lipid utilization influences regenerative competence. Transient overexpression of acid ceramidase (AC) converts ceramide into sphingosine conferring durable post-MI cardio protection and modulating apoptosis and metabolic patterns [322, 323]. Compared with that in P1 hearts, the expression of omega-3 derivative 12-HEPE is significantly reduced in postnatal P7 hearts and promotes regeneration by inhibiting Hippo signaling pathway, activating glycolysis and inducing histone H3K18 lactylation [324]. Ketone metabolism introduces an additional axis of regulation. β-hydroxybutyrate supplementation improves myocardial energetics and modulates chromatin, linking ketone metabolism to epigenetic accessibility and regenerative transcription [143, 325, 326]. FAO suppression, rebalancing of sphingolipid activity and ketone regulation converge to reduce metabolic maturation pressure and reopen plasticity features characteristic of the neonatal myocardium.

Beyond these foundational interventions, more targeted substrate-modulating approaches seek to optimize myocardial fuel selection and energy efficiency. Agents such as trimetazidine and perhexiline partially inhibit long-chain FAO, thus increasing glucose oxidation and ATP yield per unit oxygen, and have been associated with improvements in left-ventricular function and symptoms in selected patients with ischemic heart disease or HF in randomized studies summarized in metabolic-therapy reviews [195, 319, 320, 327]. As the understanding of myocardial metabolic plasticity deepens, strategies that explicitly aim to enhance myocardial repair and potentially regeneration through metabolic support are entering early clinical exploration. Small clinical and translational studies of exogenous ketone administration suggest that therapeutic ketosis can acutely improve hemodynamics and indices of cardiac energetics in HF, while optimized metabolic support regimens, including carefully titrated glucose–insulin–potassium approaches, have shown signals of benefits in ischemia–reperfusion settings in older randomized and mechanistic trials summarized in contemporary reviews [328–331]. Although still investigational, these metabolically oriented strategies reflect an emerging paradigm in which metabolic modulation is leveraged not only to protect the injured myocardium and correct energetic deficits, but also to improve cardiomyocyte survival, limit fibrosis, and provide a more permissive substrate for regenerative interventions such as gene modulation, metabolic reprogramming of cardiomyocytes and cell-based therapies.

#### Targeting mitochondrial function and redox homeostasis in cardiac repair and regeneration

Mitochondria act as integrators of substrate use, apoptosis and oxidative stress. Doxycycline, which inhibits mitochondrial ribosomal translation, promotes cardiomyocyte proliferation and improves post-MI function in adult mice via eIF2α/ATF4/KNL1 signaling [332]. Enhanced mitochondrial quality control also yields regenerative benefits: rapamycin-induced mitophagy improves survival and reduces ROS accumulation after ischemic injury in neonatal mice and zebrafish AR models [333, 334]. The mitochondrial regulator RGFP966 suppresses HDAC3, increases mitophagy and limits apoptosis after LAD ligation, whereas the HDAC3 agonist ITSA-1 reverses these benefits [335–337].

Excessive mitochondrial fission impairs cardiac regeneration capacity. Pharmacological inhibition of mitochondrial fission using Mdivi-1 mitigates pathological mitochondrial fragmentation, reduces ROS levels and improve cardiac survival after I/R injury [338]. Redox-targeted therapeutics including Coenzyme Q10 act as mitochondrial antioxidants and preserve energy in injured myocardium to create a pro-regenerative environment [339, 340]. Mitochondrial translation control, mitophagy tuning and ROS mitigation jointly reduce stress-induced cell-cycle blockade and create a more permissive bioenergetic state.

NAD⁺ depletion is characteristics MI and chronic HF. In rodents and early human studies, supplementation with nicotinamide riboside increases NAD⁺ bioavailability, improves mitochondrial function and reduces maladaptive remodeling [341–343]. Mechanistically, NAD⁺ fuels sirtuins and PARPs, linking metabolism to chromatin state and DNA-damage responses. NAD⁺ repletion does not appear to directly drive proliferation but rather primes the mitochondrial and chromatin environments required for downstream regenerative interventions.

### Cell based therapies for cardiac repair and regeneration

Reprogramming strategies represent a regenerative approach that operates at the level of cell‐fate and developmental-stage control, introducing a new direction for heart regeneration research. Unlike approaches such as cell transplantation, tissue engineering, or paracrine-mediated repair, reprogramming strategies seek to directly modify cellular identity or maturation status within injured cardiomyocytes that convert resident nonmyocytes or even mature cardiomyocytes into cells capable of contraction and electrical conduction. As a result, the therapeutic target shifts from supplementing exogenous cells to redefining endogenous cellular lineages and states, enabling the potential in situ rebuilding of contractile tissue at the organ level.

In the domain of fibroblast-to-induced cardiomyocyte (iCM) conversion, Ieda et al. identified the transcription factor combination Gata4, Mef2c, and Tbx5 (GMT) through systematic screening as sufficient to directly reprogram fibroblasts derived from mouse cardiac and dermal sources into cardiomyocyte-like cells in vitro [233]. GMT-induced cells upregulated cardiac structural and contractile genes, assembled organized sarcomeres, and demonstrated spontaneous contractile activity, indicating that cross-lineage direct reprogramming can generate cardiomyocyte-like cells without passing through a pluripotent state. Zhao et al. subsequently augmented this cocktail by incorporating Hand2 (GHMT), which increased reprogramming efficiency and resulted in induced cells with more structured sarcomere organization and more stable Ca^2^⁺ handling, showing that optimization of transcription factor composition can further enhance the functional and maturation features of iCMs [344].

Evidence from in vitro systems was later extended to in vivo cardiac reprogramming. In a myocardial infarction (MI) model established by ligating the left anterior descending artery, Qian et al. injected high-dose GMT retroviral vectors into the infarct border zone and used *Postn*-Cre or *Fsp1*-Cre lineage labeling to trace cardiac fibroblasts [344]. Approximately 15% of GMT-transduced fibroblasts in the injured region expressed α-actinin + and cTnT +, formed sarcomeric structures, and exhibited cardiomyocyte-like electrical activity and functional coupling. At 8–12 weeks of follow-up, the left ventricular ejection fraction, stroke volume, and cardiac output were greater in mice that receiving GMT, accompanied by smaller scar area and increased vascular density. Subsequent studies have employed multi-factor, multi-vector approaches and polycistronic constructs (MGTH2A) to increase co-transduction efficiency and further augment improvements in ejection fraction and scar regression [345]. Non-integrating vectors like Sendai virus were used for local delivery of reprogramming factors in rodent hearts and similarly reduced collagen deposition and improved contractile parameters [346]. Multiple independent researchers have reproduced key findings that including fibroblast-to-iCM conversion, fibrosis reduction, and improved ventricular remodeling in mouse MI models [347]. Tani et al. applied modified reprogramming cocktails across models of acute MI and chronic cardiomyopathy and demonstrated reduced wall thinning, decreased fibrosis, and functional improvement, thus extending the applicability of in vivo reprogramming from acute injury repair to chronic structural myocardial disease [348].

Beyond traditional reprogramming via viral delivery of cardiac-specific transcription factors, small molecule-based chemical reprogramming has emerged as a promising alternative. Fu et al. demonstrated that a cocktail of six small molecules named CRFVPT (CHIR99021, RepSox, Forskolin, VPA, Parnate, and TTNPB) could successfully reprogram mous fibroblasts into beating iCMs [349]. These chemically induced iCMs expressed canonical cardiomyocyte markers (Mef2c, cTnT, Gata4, α-MHC, and α-actinin) and exhibited characteristic electrophysiological properties. Notably, unlike fibroblasts undergoing GMT-based direct reprogramming, fibroblasts undergoing chemical reprogramming were found to pass through a cardiac progenitor stage. Chemical reprogramming represents potential that translating human somatic cells to CiCMs (chemically induced CMs) which requires a nine-molecule cocktail known as 9 C (comprising CHIR99021, A83-01, BIX01294, AS8351, SC1, Y27632, OAC2, SU16F, and JNJ10198409) [350]. Compared with transcription factor-based protocols, the 9 C chemical approach yields significantly higher conversion efficiency, resulting in up to 97% of CiCMs exhibiting spontaneous beating. Furthermore, the transplantation of 9C-treated human foreskin fibroblasts into infarcted murine hearts resulted in in vivo iCM generation, suggesting that the targeted delivery of the 9 C cocktail to cardiac fibroblasts holds significant therapeutic potential for myocardial infarction repair.

Complementing cross-lineage conversion strategies, recent efforts have focused on lineage-intrinsic reprogramming within mature cardiomyocytes. By utilizing a cardiomyocyte-specific and doxycycline-inducible system, the short-term expression of the Yamanaka factors (Oct4, Sox2, Klf4, and c-Myc) in adult mouse hearts successfully induced the fetal-like state [147]. Phenotypically, these cells underwent sarcomere disassembly and cell size reduction. Transcriptional analysis revealed the reactivation of fetal cardiac programs alongside the suppression of adult structural and fatty acid oxidation gene networks. Consequently, the cardiomyocytes re-entered the cell cycle and displayed proliferative characteristics reminiscent of the neonatal myocardium. In myocardial infarction (MI) models, transient induction around the time of injury reduced infarct size, improved the left ventricular ejection fraction, and enhanced hemodynamics [147]. These findings suggest that temporarily resetting the developmental state enables adult cardiomyocytes to facilitate tissue regeneration. However, prolonged factor expression drove excessive dedifferentiation toward pluripotency and caused teratoma-like lesions, underscoring the critical need for precise temporal and dosage control in myocardial rejuvenation strategies.

Collectively, these findings suggest that cardiac reprogramming advances heart regeneration along two mechanistic trajectories: cell lineage conversion and developmental-stage reversal. By redirecting fibroblasts toward an induced CM fate and restoring the proliferative capacity of mature cardiomyocytes, experimental evidence has demonstrated that new myocardia can be generated in vivo from endogenous cell sources. Findings across in vitro platforms, in vivo infarction models, lineage-tracing studies, and vector-engineering frameworks establish a continuous body of evidence showing that, even without exogenous stem-cell delivery, engineering the endogenous cell fate can modulate post-injury remodeling at both the structural and functional levels.

### Gene editing/RNA-based therapies for cardiac repair and regeneration

Non-coding RNAs (ncRNAs) and derived interventional strategies have evolved from basic transcriptional targets into core components of cardiac therapy. Accumulating evidence indicates that diverse ncRNAs not only drive essential pathophysiological processes but also systemically orchestrate the post-injury transcriptional and signaling profiles via multigene networks.

#### Virus vectors

The translational progress of RNA and gene therapies in cardiovascular medicine is heavily contingent upon delivery efficiency and spatiotemporal control [351, 352]. Consequently, adeno-associated viruses (AAVs) and other high-efficiency transduction vectors have emerged as the benchmark delivery platforms for both basic research and preclinical trials [352–355]. Liu et al. demonstrated in a porcine model of ischemic myocardial injury that AAV9-mediated Sav1 shRNA delivery promotes cardiomyocyte cell-cycle re-entry, reduces scar formation, and improves left ventricular systolic function. Mechanistically, this strategy relieves Hippo-mediated repression of YAP, thereby inducing cardiomyocyte renewal and repair responses, providing key large-animal preclinical evidence for RNAi-based gene therapy aimed at endogenous cardiac regeneration [259]. Based on these research outcomes, AAV9-Sav-shRNA (YAP101) has progressed to clinical evaluation as a first-in-class genetic medicine (NCT06831825), leveraging the AAV9 vector as an efficient delivery platform to induce endogenous cardiac regeneration.

Among the diverse repertoire of non-coding RNAs, circular RNAs (circRNAs) have distinguished themselves through their exceptional stability and regulatory potential [356]. First, regarding their attributes as therapeutic candidates, circular RNAs (circRNAs) exhibit prominent translational advantages over linear RNAs [357]. Formed by a covalently closed continuous loop via back-splicing, circRNAs naturally lack free 5’and 3’ends, rendering them highly resistant to exonuclease degradation and granting them an extended half-life compared to linear transcripts [358]. Crucially, this remarkable intracellular longevity, combined with their compact sequence requirements, makes them ideal engineered payloads for viral vectors [359]. When coupled with adeno-associated virus (AAV) delivery systems, circRNAs can achieve sustained and effective therapeutic concentrations within the myocardium [360]. Furthermore, circRNAs demonstrate robust tissue specificity and responsiveness to pathological conditions, which significantly enhances their potential as both therapeutic targets and payloads in cardiovascular medicine [361].

Exemplifying this AAV-circRNA therapeutic synergy, circHipk3 emerges as one of the most validated pro-regenerative circRNAs. Si et al. discovered that circHipk3 is upregulated during the regeneration-associated phases in a murine MI model [362]. Subsequent investigations utilizing AAV9-mediated intra-cardiac overexpression confirmed that circHipk3 significantly increases the proportions of Ki67 + and pH3 + cardiomyocytes, reduces TUNEL + cells, decreases fibrotic area, and ultimately improves cardiac function. Mechanistically, circHipk3 simultaneously interacts with Notch1 and miR-133a to orchestrate cardiomyocyte proliferation alongside the proliferation, migration, and tube formation of coronary endothelial cells. This synergistic mechanism endows circHipk3 with the unique capacity to drive the dual regenerative programs of remuscularization and revascularization [362]. Further highlighting the utility of AAV platforms in unlocking circRNAs as potent therapeutic agents, circCDYL represents another distinct class of pro-reparative circRNAs with substantial therapeutic promise. Zhang et al. reported a significant downregulation of circCDYL following acute MI model [363]. In vivo overexpression of circCDYL via AAV9 vectors successfully augments cardiomyocyte proliferation within the infarcted heart, leading to improved left ventricular function and attenuated tissue injury. This study elucidates the role of circCDYL as a crucial competing endogenous RNA (ceRNA) node, which functions by sponging downstream miRNAs to relieve their repressive effects on regeneration-associated genes, thereby potentiating myocardial repair [363].

#### Extracellular vehicles and exosomes

Although significant progress has been made in stem cell-based heart repair, persistent myocardial regeneration in adult mammals remains difficult to achieve. Recent mechanistic and lineage-tracing evidence has challenged the traditional paradigm that cell transplantation directly ameliorates the injured heart. In contrast, current evidence suggests that the improvement of cardiac function is mainly due to factors that regulate the host microenvironment rather than transplanted cells alone. Accumulating evidence from MI models suggests that the functional benefits derived from MSC or iPSC-CM transplantation are largely attributable to paracrine effects rather than direct cell replacement [364]. This implies that extracellular vehicles (EVs) secreted by these highly proliferative cells may serve as the primary functional units. This insight has inspired a paradigm shift toward cell-free therapy [365], a strategy aimed at recapitulating the therapeutic benefits of cell therapy while circumventing complications inherent to live cell transplantation [54, 366], such as immune rejection and arrhythmia [367].

Cardiomyocyte-derived EVs (CM-EVs) have been demonstrated to extensively recapitulate the paracrine benefits of parental cell transplantation. By shuttling a network of anti-apoptotic, anti-fibrotic, and pro-angiogenic miRNAs, CM-EVs preserve mitochondrial homeostasis via mTOR signaling inhibition and autophagy enhancement, thereby significantly ameliorating ischemic injury and improving left ventricular ejection fraction [368]. Unlike direct cell transplantation, CM-EV therapy circumvents arrhythmogenic risks and has broad targeting capabilities across endothelial cells, fibroblasts, and immune cells [369]. Concurrently, EVs derived from cardiac progenitor cells (CPC-EVs) and cardiosphere-derived cells (CDC-EVs) have demonstrated robust regenerative potential [369]. Studies by Barile, Emmert, and others indicate that human CPC-EVs significantly reduce infarct size in both acute and chronic myocardial infarction models, including large animals [370, 371]. Additionally, ticagrelor (the conventional drug used in infarcted patients) increases the release of CPC-EVs by increasing their proliferation rate, which promotes effective cardiac repair [370, 372].Mechanistically, CDC-EVs (enriched in miR-146a) [373] and CPC-EVs facilitate the polarization of macrophages from a pro-inflammatory M1 phenotype to a reparative M2 phenotype, effectively resolving the inflammatory microenvironment [374]. In terms of clinical translation, CPC-EVs have shown preliminary safety and efficacy in patients with non-ischemic cardiomyopathy [375]. Strategies such as hypoxic preconditioning can further enrich stress-responsive miRNAs within their cargo, potentiating therapeutic outcomes [376].

Cardiosphere-derived cells (CDCs) constitute a heterogeneous population of cardiac-derived cells generated through a multiphase culture process involving explant outgrowth, cardiosphere formation, and subsequent replating. The therapeutic efficacy of CDCs is predominantly governed by paracrine mechanisms. Serving as the principal effectors of this paracrine cascade, CDC-derived extracellular vesicles (CDC-EVs), which encompass exosomes and small EVs, fully recapitulate the cardioprotective and reparative benefits of parent CDC therapy. Foundational work by Ibrahim et al. first established CDC exosomes as the dominant mediators of these effects, demonstrating their capacity to drive the pro-survival, pro-proliferative, and pro-angiogenic actions previously attributed to whole-cell transplantation [373]. Notably, this study identified the enrichment of miR-146a within CDC-EVs as a critical driver of cardioprotection, providing early evidence for the functional contribution of exosomal microRNAs to myocardial repair. The translational relevance of CDC-EVs was subsequently validated in a large-animal setting by Gallet et al., who demonstrated that CDC exosome administration effectively reduced scar burden, blunted adverse remodeling, and preserved ventricular function in a porcine model of myocardial infarction [377]. Providing further mechanistic depth, de Couto et al. identified miR-181b as a key regulator of macrophage polarization via the repression of PKCδ [374]. This crucial finding established immunomodulation and inflammatory reprogramming as central pillars of CDC-EV-mediated cardiac repair.

Mesenchymal stem cell-derived EVs (MSC-EVs) represent the most extensively characterized cell-free therapeutic agents, with seminal work by Lai et al. establishing them as the primary effectors of MSC-mediated paracrine protection [378]. Whether derived from bone marrow or adipose tissue, MSC-EVs contain large amounts of cardioprotective molecules such as miR-21a-5p and miR-125b-5p, which effectively mitigate oxidative stress, apoptosis, and fibrosis [379]. Notably, EVs derived from hypoxia-preconditioned MSCs upregulate the expression of pro-proliferative factors such as miR-210; upon uptake by recipient cardiomyocytes, compared to their normoxic conditions, these EVs activate endogenous Sca-1⁺ cardiac stem cells, resulting in superior regenerative capacity compared to their normoxic conditions [380]. Furthermore, MSC-EVs excel in modulating local inflammation by downregulating the expression of pro-inflammatory cytokines such as IL-1β, IL-6, and TNF-α, and promoting macrophage phenotype switching, thereby establishing a microenvironment conducive to myocardial repair [381].

EVs derived from pluripotent stem cells and their derivatives (Pluri-EVs) are increasingly viewed as potential therapeutics that may surpass traditional adult stem cell-derived EVs [382]. Comparative studies by González-King et al. revealed that compared to CM-EVs, CPC-EVs, and MSC-EVs, Pluri-EVs exhibit superior efficacy in promoting angiogenesis, inhibiting fibrosis, and reversing pathological remodeling [383]. Even under high-stress conditions like I/R models, Pluri-EVs maintain robust cardioprotection, likely attributable to their cargo enriched with developmental and regeneration-associated signals [384, 385]. Compared with the tumorigenic and immunogenic risks associated with live pluripotent stem cell transplantation, Pluri-EVs offer a safer alternative, demonstrating greater efficacy in reducing post-infarction collagen deposition and α-smooth muscle actin-positive myofibroblasts, positioning them as highly promising candidates for MI treatment [386].

As pivotal mediators of cellular communication, specific EVs play regulation roles in physiological and pathological conditions. Developmentally, placenta-derived EVs have been shown to promote cardiomyocyte maturation and facilitate fetal heart development [387]. Insights from developmental biology and bioengineering are shaping the future of EV therapeutics. Research indicates that EVs derived from regenerative neonatal hearts (AR-Neo-EVs) carry specific proteins such as Wdr75, which can release the proliferation block in adult cardiomyocytes [388]. Conversely, EVs released during post-natal maturation suppress regeneration via the Pak2-Erk1/2 pathway, highlighting the decisive role of environmental EVs in toggling regenerative potential [389]. Evolutionary evidence further suggests that EVs secreted by highly regenerative species, such as newts, confer protection to mammalian cardiomyocytes, implying the interspecies conservation of key regenerative signals [390, 391]. On the application front, engineering strategies have significantly enhanced EV efficacy: for instance, thymosin β4-loaded biomimetic EVs demonstrate precise homing to infarcted regions, while shock wave therapy (SWT) induces the release of miR-19a-3p-enriched EVs from endothelial cells, effectively promoting neovascularization [392, 393]. These approaches, integrating developmental insights with molecular engineering, are paving new avenues for cell-free therapies in myocardial infarction.

During acute MI, a surge of EVs derived from damaged cardiomyocytes, fibroblasts, and endothelial cells can promote pro-inflammatory cytokine production in infiltrating monocytes, thereby exacerbating local inflammation [394]. While the pathogenic roles of EVs in MI have been extensively reviewed elsewhere, it is established that under physiological conditions or therapeutic manipulation, EVs carry cardioprotective cargo. Given that EVs from diverse cellular sources have demonstrated robust regenerative effects in preclinical models, this section will focus on the therapeutic application of EVs in orchestrating cardiac repair and regeneration [395, 396].

#### Delivery routes in gene editing/RNA-based therapies

Systemic intravenous delivery remains severely restricted by hepatic sequestration and reticuloendothelial clearance, yielding sub-therapeutic myocardial accumulation [351]. Localized administration such including intramyocardial injection, intracoronary administration and pericardial administration is biologically necessary to direct RNA agents specifically to the heart [397, 398].

In cardiac RNA therapeutics, the administration route constitutes a primary determinant of both efficacy and safety. This significance stems from the fact that the delivery pathway directly dictates pharmacokinetics, tissue distribution, cellular uptake, and expression kinetics [397, 399, 400]. Ultimately, precise control over these delivery-dependent variables dictates the therapeutic viability of RNA agents within the complex myocardial architecture.

At the macroscopic level of systemic distribution, different delivery routes govern the ultimate organ fate of RNA agents. Under current technological constraints, intravenous administration frequently leads to the sequestration of nucleic acids within the liver and reticuloendothelial system, yielding sub-therapeutic myocardial accumulation. Conversely, local approaches such as intracoronary or intramyocardial administrations significantly enhance regional cardiac exposure, though they inherently differ in perfusion range, therapeutic duration, and spatial distribution [400]. Furthermore, pericardial administration offers an intermediate strategy maximizing payload retention at the epicardial surface while the penetration may remain more limited than with direct intramyocardial deposition [398, 401]. Therefore, selecting a delivery route fundamentally represents a choice among distinct spatial distribution patterns rather than a mere technical substitution. Beyond organic targeting, the delivery pathway determines the cellular compartments within the heterogeneous cardiac microenvironment. The heart comprises diverse populations including cardiomyocytes, fibroblasts, endothelial cells, and immune cells, and the administration route dictates the preferential uptake of RNA molecules by these distinct lineages. For instance, intracoronary delivery facilitates greater interaction with endothelial and circulating cells [402]. Carlsson et al. demonstrated the therapeutic efficacy of VEGFA-modRNA in a porcine myocardial infarction model, establishing this intramyocardial approach as a landmark clinical-stage gene therapy for targeted cardiac repair [403]. Conversely, intramyocardial injection affords direct access to in situ cardiomyocytes. Gallet et al. demonstrated that the intracoronary administration of CDC exosomes effectively delivers cardioprotective microRNAs, notably miR-146a, to the injured myocardium, thereby reducing scar burden, attenuating adverse ventricular remodeling, and improving overall cardiac function in a porcine model of MI [377]. Concurrently, pericardial administrations preferentially modulate epicardial and immune responses [404]. This defined cell-type tropism is crucial, as therapeutic objectives like myocardial regeneration, fibrotic attenuation, and immune reprogramming fundamentally depend on engaging entirely different target cell populations.

Finally, regarding expression kinetics and the therapeutic window, the delivery pathway acts synergistically with the chosen vector to define the peak exposure and functional duration of the RNA payload. Regenerative interventions typically necessitate brief and strictly controlled expression windows. Localized, high-concentration delivery methods can generate the rapid onset and controlled decay required for these transient stimuli. In contrast, systemic administration or viral vector-mediated delivery often results in prolonged expression profiles, which may introduce risks such as sustained cellular dedifferentiation and arrhythmogenesis. Consequently, the administration route actively defines the temporal dimension of the therapy, an aspect of paramount importance in the field of cardiac regeneration.

### Biomaterial based strategies for cardiac repair and regeneration

In the field of cardiac regeneration and repair, the direct administration of isolated cells or nucleic acids is frequently hindered by severe translational bottlenecks, including low target-site retention, rapid systemic clearance, and uncontrollable expression kinetics. The introduction of biomaterials offers a foundational strategy to overcome these limitations. In recent years, chemical scaffolds, such as injectable hydrogels and cardiac patches, have been combined with micro- and nanoscale delivery vehicles based on nanoengineered biomaterials. Together, they establish a comprehensive multiscale platform for targeted therapy.

#### Injectable hydrogels and cardiac patches

Precise modulation of the cellular microenvironment is a pivotal determinant of cell fate and function. As a fundamental component of this microenvironment, the extracellular matrix (ECM) provides critical biochemical and biophysical cues. However, traditional strategies involving direct cell injection often fail to achieve optimal viability and functional integration due to the absence of supportive cell–matrix interactions. In the context of tissue engineering, biomaterials offer an ideal avenue to recapitulate ECM-like physicochemical interactions, thus potentiating the retention, survival, and functionality of both transplanted and recruited cardiac-lineage cells. Among various biomaterials, hydrogels—composed of natural or synthetic hydrophilic, biocompatible polymer networks—serve as promising vehicles for cell and drug delivery, as well as platforms for elucidating the biology of cardiac-derived stem cells. A defining feature of hydrogels is their capacity for in situ gelation from aqueous solutions, which provides a mechanism for the injection and subsequent immobilization of cell/polymer suspensions upon reaching the target site in the infarcted heart. This injectability confers a distinct advantage upon hydrogels over other biomaterial forms, such as solid scaffolds, particularly for minimally invasive cardiac cell therapy. To date, a diverse array of polymers, including collagen, fibrin, alginate, polyethylene glycol (PEG), and self-assembling peptides, have been extensively evaluated for their efficacy in forming hydrogels for cardiac tissue engineering applications [405].

Injectable hydrogels have been used to reinforce infarcted myocardium and deliver pro-regenerative cues. Wang et al. reported that a hyaluronic-acid containing hydrogel carrying miR-302 was used to induce cardiomyocyte cell-cycle re-entry and improve ventricular function after myocardial infarction in mice [210]. Li et al. used an alginate-based microgel system to deliver AR-Neo-EVs to enhance cardiomyocyte proliferation and angiogenesis in infarcted mouse hearts [388]. Another study facilitates the alginate-based BIO-IGF-1 co-loaded gelatin nanoparticles (CgNPs) promoted regeneration hallmarks and cardiac functions in rat MI models [406]. Four additional studies investigated the efficacy of hydrogel derivatives across multiple species MI models. These interventions were shown to augment post-infarction repair and ameliorate cardiac function, effects that were mechanistically attributed to the inhibition of autophagy, promotion of angiogenesis, attenuation of inflammation, and the upregulation of cardiomyocyte regeneration markers like pH3 and Ki67 [407–410].

The advent of engineered heart graft strategies is attributable to the deep convergence of biomaterials science and stem cell technology. As early as 1997, Eschenhagen et al. pioneered the engineered heart tissue (EHT) model using collagen matrices and cell gelation techniques [411, 412], thereby laying the cornerstone of this field. Prior to the use of induced pluripotent stem cell-derived cardiomyocytes (iPSC-CMs), contractile cell sources for patch construction were limited, relying primarily on primary neonatal rodent cardiomyocytes [413], with some studies attempting the use of fetal or adult cardiac cells [414]; early humanization efforts mainly depended on cardiomyocytes and progenitors differentiated from hESCs [415].

Breakthroughs in pluripotent stem cell technology have robustly rejuvenated the field, accelerating its progression toward preclinical and clinical stages. iPSC-based engineered heart tissues have demonstrated consistent functional benefits across diverse species (mice, rats and guinea pigs) and various injury models (cryoinjury, ischemia–reperfusion, and myocardial infarction), underscoring their broad biological adaptability [62, 416–421]. In 2025, the work of Jebran et al. established a new milestone that by utilizing GMP-grade engineered myocardium for allogeneic transplantation, they achieved long-term graft survival, extensive vascularization, and integrated electromechanical coupling in primates and humans [422].

The pioneering success of EHT technology in exogenous cardiac regeneration stems from its unique nature as the hybrid of engineering and biology. Unlike simple cell suspension injections, EHT not only overcomes critical bottlenecks regarding low retention and high attrition of transplanted cells but also provides immediate physical support to the damaged ventricular wall via biomimetic scaffolds, thus restricting pathological remodeling. Looking forward, the evolution of EHT is poised to transcend the concept of a simple “patch” toward sophisticated functional integration: accelerating revascularization through integrated vascular networks, optimizing electrophysiological coupling via conductive materials to minimize arrhythmic risks, and developing immunologically inert universal matrices. Consequently, EHT technology is progressively bridging the “translational gap”, positioning itself as one of the most promising therapeutic avenues for end-stage heart failure.

#### Nanoengineered biomaterials

Zheng et al. reported that pomegranate-polyphenol–derived biogenic nanoparticles were used to modulate mitochondrial membrane potential, reduce ROS levels and improve contractility in engineered heart tissues and mouse myocardium, thus increasing cardiomyocyte survival under injury [423]. Aoyama et al. used ultraflexible mesh nanoelectronics to record electrical activity from transplanted human cardiomyocytes and reveal ectopic firing and reentry circuits in vivo [424]. In the same platform, the self-assembling peptide RADA16 was used as an injectable nanofiber hydrogel to encapsulate hiPSC-cardiomyocytes, improve graft retention, stabilize electrical coupling and reduce graft-induced ectopic activity [424]. Together, these nanoengineered antioxidant particles, soft electronic meshes and peptide hydrogels were used to regulate mitochondrial homeostasis, support graft organization and enable electrophysiological monitoring, creating conditions that facilitate cardiomyocyte engraftment and regeneration.

In summary, exogenous reprogramming represents a precision medicine strategy that aims to activate dormant proliferation programs within a transient regenerative window through the efficient delivery of vehicles and the targeted efficacy of their cargos. In addition to its high translational feasibility and potential for standardized manufacturing, the method circumspect the complexity inherent in the introduction of foreign cells. The flourishing of this field is driven by the resonance between modern biomedicine and advanced materials science. As a profound integration of these disciplines, exogenous reprogramming is evolving in a more intelligent and biomimetic direction, with numerous unknown areas such as surface modification of nanocarriers and temporal regulation of genes attracting future research.

## Challenges in the clinical translation of cardiac regenerative and repair therapies

Although progress has been made in the research and application of cardiac regeneration therapies, significant challenges remain in translating animal studies to clinical practice due to interspecies differences. At the treatment strategy level, issues such as cell maturity, the timing of regenerative interventions, integration with the host heart, and long-term safety are equally important. The shortcomings in these therapies are primarily reflected in several aspects: first, the immaturity of iPSC‑CMs, which leads to functional and structural mismatches with adult cardiomyocytes; second, poor integration of transplanted cells with the host heart's electrical conduction system, resulting in arrhythmias and other electrophysiological issues; third, improper intervention timing that may lead to abnormal proliferation, affecting cardiac repair outcomes; and finally, the risks of immune rejection and abnormal proliferation, particularly when using allogeneic or reprogrammed cells. Therefore, a comprehensive understanding and resolution of these treatment limitations remain key challenges in the clinical application of cardiac regeneration therapies.

### Model-specific constrains in cardiac regeneration translation

Cardiac regeneration varies markedly across species, reflecting differences in cardiomyocyte biology, metabolic states, immune dynamics and extracellular-matrix properties. Highly regenerative species such as zebrafish and urodele amphibians maintain predominantly mononuclear, diploid cardiomyocytes with intact centrosomes and relatively immature sarcomere architecture, features that permit efficient cell-cycle re-entry after injury [47]. Their regenerative responses are further characterized by cardiomyocytes that remain in a metabolically flexible, relatively glycolytic state with low mitochondrial reactive oxygen species (ROS) burden, conditions associated with preserved proliferative competence [425]. By contrast, adult mammalian cardiomyocytes undergo rapid postnatal maturation characterized by a metabolic switch from glycolysis to fatty-acid oxidation. This transition increases mitochondrial oxidative load and ROS generation, activating DNA-damage responses that enforce cell-cycle exit and promote a stable post-mitotic state [45]. The concurrent acquisition of binucleation and polyploidy further restricts proliferative potential and correlates with diminished regenerative capacity in adult mammalian hearts [47]. Differences in immune responses also contribute: regenerative species mount rapid, self-limited inflammatory reactions that support cardiomyocyte proliferation and matrix remodeling, whereas mammals exhibit more prolonged, CCR2⁺ monocyte-derived macrophage-dominated inflammation that favors fibrosis and adverse remodeling [35, 425]. Vascular and extracellular-matrix dynamics represent additional cross-species determinants. Regenerative vertebrates rapidly revascularize injured myocardium and deploy lymphatic networks that facilitate clearance of necrotic debris and resolution of inflammation, whereas these processes are slower and less efficient in adult mammals [425]. Extracellular-matrix composition and stiffness also differ: zebrafish and amphibians maintain a compliant, dynamically remodeled matrix that can regress as muscle mass is restored [426–429], while the mammalian myocardium tends to form a dense, mechanically stiff scar that stabilizes the ventricle but effectively locks cardiomyocytes in a non-proliferative state [284, 430, 431]. Collectively, these cross-species comparisons define a mechanistic spectrum governing vertebrate cardiac regeneration and link structural, metabolic and immunological traits to regenerative outcome.

Despite the mechanistic insights gained from regenerative species, substantial biological barriers hinder direct translation to adult mammals. Mitochondrial architecture represents a key constraint that zebrafish and amphibian cardiomyocytes contain small, structurally simple mitochondria optimized for relatively low oxidative demands [20, 427], whereas adult mammalian cardiomyocytes harbor large, densely packed mitochondria with complex cristae and high oxidative capacity [45, 432]. Comparative analyses of mitochondrial structure and function across commonly used preclinical species indicate that the adult mammalian mitochondrial network reinforces sustained oxidative phosphorylation and high ROS production [100, 156, 432]. These conditions actively suppress cardiomyocyte proliferation and favor hypertrophic rather than hyperplastic growth [433].

Inflammatory and extracellular-matrix dynamics further restrict translation. Regenerative species exhibit tightly timed inflammatory transitions with rapid resolution and reversible matrix remodeling, whereas adult mammals sustain prolonged inflammation and stable collagen deposition that prevent cardiomyocyte re-entry into the cell cycle [35, 66, 434]. Structural limits also arise from differences in vascular and lymphatic systems: compared with zebrafish and amphibians, mammalian hearts show slower angiogenesis and less efficient lymphatic clearance after injury, which constrains the window during which a pro-regenerative niche can be maintained [425, 435, 436].

These model-specific constrains underscore a central challenge in cardiac regeneration research: regenerative pathways that operate successfully in fish or amphibians are embedded in biological contexts that are fundamentally different from the metabolic, structural and immunologic milieu of the adult mammalian myocardium. As a result, successful translation will require not simple pathway transfer but systematic strategies that re-engineer the mammalian cellular environment at the levels of cardiomyocyte ploidy and metabolism, mitochondrial function, immune responses and matrix mechanics to approximate the key features of naturally regenerative hearts.

### Challenges of metabolic derangements

Metabolic targets, including CPT1, PDK, and key glycolytic regulators, offer distinct mechanistic entry for the reactivation of cardiomyocyte plasticity and proliferation. However, the clinical translation of these targets is complicated by the integrated system of metabolic networks, where targeted interventions often elicit broader physiological consequences.

For instance, the dose-related hepatotoxicity observed with the CPT1 inhibitor etomoxir in clinical cohort underscores liver injury and oxidative stress burden imposed by the systemic inhibition of fatty acid oxidation [318, 437]. The pharmacological inhibition of PDK by dichloroacetate (DCA) facilitates glucose oxidation; however, its clinical development has been hindered by adverse effects, most notably peripheral neurotoxicity [438]. Such outcomes suggest that the constitutive redirection of mitochondrial substrate preference entails significant biological costs. Furthermore, the direct inhibition of glycolysis with 2-DG frequently results in systemic energy stress and poor tolerability [439, 440]. These findings indicate that without precise spatiotemporal control, metabolic interventions are difficult to sustain within a viable therapeutic window. Consequently, the translation of metabolic strategies for cardiac regeneration necessitates evidence beyond the mere improvement of cardiac function. It is imperative to demonstrate that localized metabolic flux reprogramming effectively drives regenerative endpoints, such as cytokinesis and myocardial formation, while simultaneously characterizing the boundaries of systemic metabolic disruption and organ toxicity. A comprehensive assessment of these parameters is essential for ensuring the safety and feasibility of such interventions.

### Challenges of tumorigenesis and maturity deficiency

The fundamental objective of cardiac regenerative medicine is to reactivate the proliferative and plastic potential of adult cardiomyocytes. However, this therapeutic goal is inherently intertwined with the molecular mechanisms of oncogenesis, making tumorigenic risk a structural constraint rather than a peripheral concern. Previous research indicates that this risk manifests as direct neoplastic phenotypes, the loss of control over cellular plasticity during in vivo reprogramming, and a profound mechanistic overlap between pro-regenerative and classical oncogenic networks.

The most direct evidence of oncogenic risk stems from transplantation models involving iPSCs. Early animal studies demonstrated that undifferentiated ESCs or iPSCs can form teratomas after intramyocardial injection. Buccini et al. reported that the presence of undifferentiated cells leads to cardiac teratoma formation in mice, which identifies residual pluripotent cells as the most immediate source of tumor risk in cell therapy [441]. Subsequently, preclinical safety evaluations conducted by Ito and colleagues highlighted that even trace amounts of undifferentiated cells pose potential oncogenic hazards [442]. These studies collectively emphasize the urgent need for high-sensitivity detection systems and strict thresholds for residual cell counts to ensure clinical safety.

Maturity deficiency in iPSC‑CMs is another core challenge in cardiac regeneration therapies. Although iPSCs can differentiate into cardiomyocyte-like cells, these iPSC‑CMs often exhibit phenotypic and functional differences when compared to adult cardiomyocytes, which limits their therapeutic potential in post-injury heart repair. iPSC‑CMs typically demonstrate structural and functional immaturity, lacking key components such as striated muscle fibers, a mature T-tubule system, and properly organized sarcomeres, which are critical for normal contraction and electrophysiological properties. Furthermore, iPSC‑CMs tend to rely on glucose metabolism (glycolysis) rather than FAO, the main energy source in mature cardiomyocytes. This metabolic immaturity reduces their energy efficiency and limits their ability to perform at the level of adult heart cells. Addressing these deficiencies is essential for improving iPSC‑CMs' integration and functionality within the damaged myocardium, thereby enhancing the efficacy of cardiac regeneration strategies [443, 444].

Beside the riskily direct transplantation of undifferentiated cells, regenerative strategies may still encounter complex boundaries of cellular plasticity. In vivo reprogramming research provides a critical warning in this regard. Abad et al. demonstrated that the systemic induction of OSKM factors triggers teratoma formation across multiple organs, thereby establishing a clear causal link between in vivo reprogramming and tumorigenesis [445]. This evidence suggests that tissue plasticity can rapidly shift toward malignancy once the de-differentiation program exceeds a certain regulatory threshold [446]. Although subsequent studies have attempted to achieve partial reprogramming through short-term or tissue-specific induction, comments in cardiovascular field shows that any enhancement of plasticity for regeneration must be strictly limited in duration and dosage to avoid crossing the tumorigenic threshold [447].

The potential challenge of signaling over-activated lies in the overlap between regenerative expectations and oncogenic networks. Key regulatory axes such as Hippo-YAP, PI3K-AKT, and Myc serve as essential drivers for cardiomyocyte proliferation and regeneration, but they also function as core signals in various types of cancer. For example, research by Xin and colleagues showed that YAP activation significantly promotes cardiac regeneration while YAP is widely recognized as a potent pro-cancer factor in tumor researches [262]. This molecular isomorphism implies that strategies aimed at the long-term or systemic enhancement of these pathways could theoretically increase the risk of abnormal proliferation [448]. While most current cardiac regeneration models have not yet observed clear tumor formation, many studies rely on short-term follow-up and lack data in the context of aging or metabolic disorders.

These findings suggest that the success of cardiac regeneration depends not on maximizing the intensity of proliferation but on establishing a restricted and reversible window for tissue-specific growth. Future preclinical research should include long-term tumorigenicity monitoring as a standard part of safety assessments across different metabolic and age-related backgrounds.

### Challenges in intervention window and delivery methods

The timing of intervention is a critical determinant in cardiac regeneration therapy, as it directly affects the controllability of cell proliferation. Regenerative therapies must be applied within a specific time window after injury to effectively activate the regenerative potential of cardiomyocytes by utilizing metabolic, genetic, or transcriptional factors. Intervening too early or too late can increase the risk of uncontrolled proliferation, which compromises the regenerative process.

Delayed intervention results in the loss of the regenerative window and the progression to fibrosis, severely limiting the potential for effective tissue repair [9, 69]. In addition to impairing regeneration, inappropriate timing can lead to abnormal cell proliferation. For example, in cardiac reprogramming, inducing cardiomyocytes to revert to a fetal-like state can promote repair, but if performed outside the optimal window, it may trigger excessive proliferation, leading to irregular myocardial tissue or fibrosis [147]. Such uncontrolled proliferation hinders functional integration and poses the risk of tumor formation. Therefore, precise control of intervention timing is crucial to optimize regenerative outcomes while minimizing the risks of abnormal cell growth.

The design of intervention timing and anatomical localization in cardiac regeneration research typically centers on the dynamic shifts of the regenerative window following myocardial injury. In neonatal mouse models, injuries are generally induced on postnatal day 1 when the heart still possesses significant regenerative capacity. Research by Porrello and colleagues using apical resection and myocardial infarction models on postnatal day 1 demonstrated that structural regeneration is achievable at this stage [9]. This specific timing has since become the gold standard for studies aiming to extend the endogenous regenerative window. In contrast, the window for effective regeneration largely closes by postnatal day 7, marking a critical transition in cardiomyocyte proliferative potential [9].

In adult mice models of acute myocardial infarction, therapeutic interventions are predominantly concentrated within the acute phase, typically ranging from a few hours to 48 h post-injury. For instance, Yang et al. administered miR-34a inhibitors at 6 h and 2 days after myocardial infarction to influence early cell fate decisions and the subsequent remodeling process [449]. The delivery methods in these small animal models often involve systemic administration through intravenous or intraperitoneal injection [449]. However, researchers also frequently employ direct intramyocardial injection into the infarct border zone to target cells that retain the highest degree of plasticity. Large animal models place a greater emphasis on clinical relevance and spatial precision during intervention. Gabisonia and colleagues utilized a porcine ischemia–reperfusion model to inject AAV-miR-199a into the infarct border zone just 10 min after reperfusion [9]. This approach specifically targets the immediate post-reperfusion window to maximize the pro-regenerative effect. For chronic injury models, Gallet et al. performed NOGA-guided injections of CDC-derived exosomes into both the scar tissue and the border zone 4 weeks after myocardial infarction [377]. This strategy allows for the evaluation of regenerative potential within a pre-existing state of adverse remodeling. In contrast to mammalian models, metabolic or pharmacological interventions in the zebrafish cryoinjury model typically begin on the first day post-injury and continue through the stages of cardiomyocyte dedifferentiation and proliferation. Various treatment methods aim to activate various regenerative processes to restore cardiac regeneration. The development of delivery methods has facilitated the rapid increase in the clinical preclinical transformation of regenerative research. By summarizing the delivery methods included in the main studies, we have drawn a more comprehensive framework to reflect the current status of cardiac regeneration research (Table 3).

Taken together, these strategies reveal a clear hierarchical structure in the timing of intervention. Neonatal studies rely on developmental windows while adult acute models focus on localized regulation within the border zone during the initial hours or days of injury. Large animal models prioritize immediate post-reperfusion or chronic remodeling phases using precision regional injections. Across all these diverse models, the preferred site of intervention remains the infarct border zone where cardiomyocyte populations are most likely to respond to regenerative cues.

### Challenge of arrhythmogenicity

A significant challenge in cardiac regeneration is the poor integration of transplanted iPSC‑CMs with the host heart's contraction and electrical conduction systems, leading to electrophysiological issues such as arrhythmias [450]. iPSC‑CMs exhibit electrophysiological characteristics that differ from those of adult cardiomyocytes, especially in terms of action potential morphology, duration, and coupling with surrounding myocardial tissue [450]. This mismatch hinders their ability to synchronize with the host heart, causing conduction abnormalities and increasing the risk of arrhythmias [451–453]. Furthermore, iPSC‑CMs may struggle to integrate effectively with the heart's native conduction system, including the sinoatrial and atrioventricular nodes, resulting in irregular heartbeats and conduction delays. This issue is particularly prominent in regions of damaged myocardium, where the risk of developing life-threatening ventricular arrhythmias is higher [454, 455]. Improving the functional integration of iPSC‑CMs with the host heart's conduction system is crucial for restoring normal cardiac rhythm and preventing electrophysiological disturbances [455, 456].

## Future perspectives

The evolutionary adaptation in the mammalian cardiac system prioritizes physiological functions at the expense of regenerative capacity, thus creating a fundamental barrier to endogenous myocardial repair. Extensive investigations have revealed molecular mechanisms that may restore cardiomyocyte proliferation. To explore human cardiac regenerative potential, researchers have established mammalian regeneration models and advanced therapeutic approaches for preclinical testing. These models indicate that mitotic activity, metabolic reprogramming, and microenvironmental remodeling collectively govern cardiomyocyte proliferation. Consequently, the integration of metabolomics, single-cell omics, and lineage tracing is imperative for identifying molecular targets and intervention strategies.

In the future development of cardiac regenerative medicine, metabolism should be repositioned as a quantifiable, predictable and intervenable key regulatory layer, rather than merely an energy background variable. An urgent question to be answered is whether specific metabolic states can predict the regenerative potential of the myocardium and guide the stratified application of metabolically targeted therapies. Metabolic profiling analysis provides a translatable tool framework for this. By systematically integrating indicators such as the ratio of glycolysis to fatty acid oxidation, NAD^+^/NADH and NADPH balance, one-carbon metabolic flux, and redox stress, it is expected to establish a "regenerative metabolic fingerprint" to distinguish individual differences that tend towards regenerative repair or fibrotic remodeling. At the preclinical level, metabolic targeted intervention needs to meet three validation criteria: Firstly, demonstrate reprogramming at the flux level rather than merely changes in steady-state concentration. Secondly, it induces a defined endpoint of regeneration, including cytokinesis and formation of new myocardium. Thirdly, maintain stability in high-risk models such as aging, diabetes and chronic heart failure. In the clinical translation stage, a stratified trial design driven by metabolic markers should be adopted, using the metabolic fingerprint as both a patient screening tool and a biomarker for drug efficacy, and linking it with imaging and structural reconstruction indicators to evaluate therapeutic efficacy. Contemporary metabolic profiling has both predictive value and monitoring function, and metabolic targeting strategies based on this development may become a realistic path for precision regenerative medicine.

Therapeutic strategies that target cardiomyocyte proliferation (specifically by enhancing glycolysis or suppressing fatty acid oxidation) have demonstrated regenerative efficacy in large-animal models. Despite promising prospects, clinical translation experiences two challenges. First, during the achievement of functional maturation and electromechanical integration of regenerated cardiomyocytes, it is necessary to simultaneously address the off-target effects and long-term safety issues of metabolic interventions. Furthermore, multidisciplinary integration is highly important for developing safer, more efficient, and personalized regenerative therapies. Future studies need to employ spatial metabolomics and multi-modal bioimaging to decipher the metabolic regulation of cardiac maturation. Moreover, the elucidation of the impact of metabolic interventions on regenerative dynamics will play a decisive role in reducing the translational risks of metabolic interventions and stem cell therapies.

## Data Availability

Not applicable.
